# Unique bacterial assembly, composition, and interactions in a parasitic plant and its host

**DOI:** 10.1093/jxb/erz572

**Published:** 2020-01-06

**Authors:** Connor R Fitzpatrick, Adam C Schneider

**Affiliations:** 1 Department of Biology, University of Toronto Mississauga, Mississauga, ON, Canada; 2 Department of Integrative Biology, University of California, Berkeley, CA, USA; 3 The James Hutton Institute, UK

**Keywords:** Holoparasite, microbiome, microbiome assembly, network analysis, Orobanchaceae, *Orobanche hederae*, parasitic plant, parasitic reduction syndrome, plant microbiome, Procrustes

## Abstract

How plant-associated microbiota are shaped by, and potentially contribute to, the unique ecology and heterotrophic life history of parasitic plants is relatively unknown. Here, we investigate the leaf and root bacterial communities of the root holoparasite *Orobanche hederae* and its host *Hedera* spp. from natural populations. Root bacteria inhabiting *Orobanche* were less diverse, had fewer co-associations, and displayed increased compositional similarity to leaf bacteria relative to *Hedera*. Overall, *Orobanche* bacteria exhibited significant congruency with *Hedera* root bacteria across sites, but not the surrounding soil. Infection had localized and systemic effects on *Hedera* bacteria, which included effects on the abundance of individual taxa and root network properties. Collectively, our results indicate that the parasitic plant microbiome is derived but distinct from the host plant microbiota, exhibits increased homogenization between shoot and root tissues, and displays far fewer co-associations among individual bacterial members. Host plant infection is accompanied by modest changes of associated microbiota at both local and systemic scales compared with uninfected individuals. Our results are a first step towards extending the growing insight into the assembly and function of the plant microbiome to include the ecologically unique but often overlooked guild of heterotrophic plants.

## Teaser

The first *in situ* investigation of bacterial communities inhabiting parasitic plants and their hosts reveals simplification and homogenization of the parasite microbiota, but also influence from the host microbiota.

## Introduction

Plants harbor rich assemblages of microorganisms, which vary in diversity and composition across host plant tissues, individuals, and species (Bulgarelli *et al.*, 2013). This variation is driven by edaphic features including the regional pool of soil microorganisms (Liu *et al.*, 2019), as well as innate plant immunity (Lebeis *et al.*, 2015; Stringlis *et al.*, 2018), the quality and quantity of plant-derived resources (Zhalnina *et al.*, 2018), and microbe–microbe interactions (Agler *et al.*, 2016; Durán *et al.*, 2018; Carlström *et al.*, 2019). Controlled experiments indicate that microbiota may play a role in plant nutrient acquisition (Castrillo *et al.*, 2017; Finkel *et al.*, 2019a) and tolerance to abiotic and biotic stress (Fitzpatrick *et al.*, 2018), including defense against pathogens (Vogel *et al.*, 2016; Berendsen *et al.*, 2018; Tsolakidou *et al.*, 2019). However, host plant species vary in the composition of their associated microbiota, and the causes and ecological consequences of this variation are poorly understood (Fitzpatrick *et al.*, 2018). Increased insight into the assembly and function of plant microbiota requires investigation of plant species that occupy diverse ecological niches (e.g. Angel *et al.*, 2016; Coleman-Derr *et al.*, 2016; Finkel *et al.*, 2016). Such an investigation provides an opportunity to address how associated microbiota are shaped by, and potentially contribute to, the functional diversity found across plant species.

**Fig. 1.**
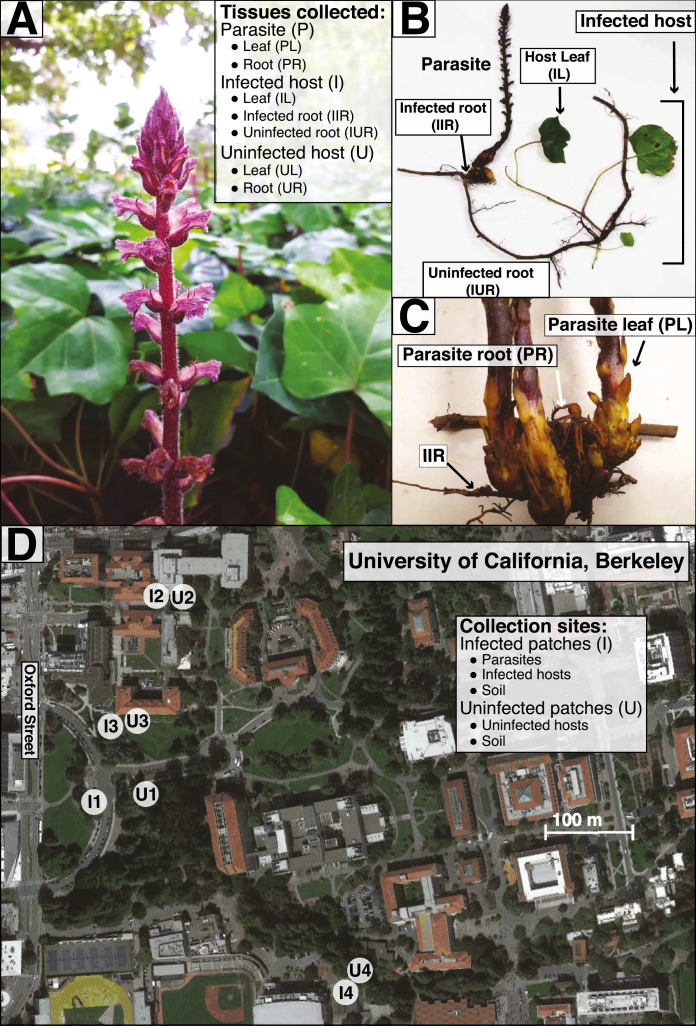
An overview of the study system and sampling design. (A) *Orobanche hederae* inflorescence in the foreground parasitizing *Hedera algeriensis* (leaves in background). (B and C) Excavated host–parasite sample showing morphology and sampled organs. For infected *Hedera*, we distinguished between infected roots (IIR), which were physically attached to the parasite, and uninfected roots (IUR), which did not exhibit direct physical attachment to any parasite. Leaves (UL) and roots (UR) were also sampled from uninfected *Hedera*. (D) Paired infected/uninfected ivy patches from the University of California campus. Abbreviations used here are consistent throughout.

One of the most dramatic niche shifts undergone by some plants is the transition from autotrophy to heterotrophy. In heterotrophic plants, resources are acquired from other vascular plants indirectly through co-associated mycorrhizal fungi (mycoheterotrophy), or directly through specialized parasitic organs called haustoria. The haustorium attaches to the root or stem vasculature of host plants and acts as a conduit through which material is exchanged primarily from host to parasite (Joel *et al.*, 2013; Yoshida *et al.*, 2016; Spallek *et al.*, 2017). Approximately 1–2% of flowering plants directly parasitize other plants to some degree in order to acquire fixed carbon, water, and/or mineral resources (Westwood *et al.*, 2010). A subset of these are holoparasites, meaning that they rely exclusively on one or more hosts for resources. Unsurprisingly, this life history shift has been accompanied by dramatic ecological and physiological shifts such as the complete loss of photosynthetic function, as well as morphological and genomic changes collectively referred to as ‘parasitic reduction syndrome’ (Colwell *et al.*, 1994). For example, root holoparasites typically exhibit vestigial, scale-like leaves, loss of a developed root system (Fig. 1A–C), and reduction in chloroplast genome size. However, very few studies have investigated changes induced in the composition and assembly of parasitic plant-associated microbial communities (Kruh *et al.*, 2017) in spite of the mounting evidence that such communities are crucial for plant function and fitness (Vogel *et al.*, 2016; Castrillo *et al.*, 2017; Fitzpatrick *et al.*, 2018).

From the perspective of the host plant, being parasitized may also perturb resident microbiota. Infected host plants have greater demands on their resources because of the significant portion of fixed carbon, water, and nutrients siphoned by the parasitic plant (Fernández-Aparicio *et al.*, 2016). Consequently, infected host plants mount physiological and molecular responses to resist the invading parasite. This can occur over both local scales (i.e. directly parasitized tissue) and systemic, whole-plant scales (Hiraoka *et al.*, 2009). These defense responses include the production of reactive oxygen species (Hegenauer *et al.*, 2016) and secondary metabolites (Serghini *et al.*, 2001; Castillejo *et al.*, 2009), as well as the activation of hormonal pathways governed by jasmonic acid and ethylene (Hiraoka *et al.*, 2009). Plant immunological responses can re-structure associated microbiota due to variation in the susceptibility of microbial taxa to plant immune outputs (Lebeis *et al.*, 2015; Stringlis *et al.*, 2018; Voges *et al.*, 2019). Finally, parasitic plant-derived molecules can alter host root growth (Spallek *et al.*, 2017), which could alter the composition of associated microbiota. Thus, by altering the quality and quantity of plant-derived resources available for microbes, activating innate plant immunity, and altering host plant morphology, parasitic plant infection could perturb the resident microbiota of their hosts.

In this study, we address three main research questions. First, what are the differences in the diversity, composition, and structure of plant microbiota between a root holoparasite and its autotrophic plant host? We predicted that parasitic plants would exhibit reduced microbial diversity and altered composition and structure relative to host plants; in other words, that the concept of the ‘parasitic reduction syndrome’ can be extended to include the microbial ecology of parasitic plants. Secondly, does the composition of parasitic plant microbiota track the composition of host plant microbiota? Due to the biased flow of material through the haustorium from host to parasite, we predicted that microbial communities associated with the parasitic plant would exhibit parallel shifts with host plant-associated communities. Thirdly, does infection by a parasitic plant result in perturbations to host plant microbiota? We predicted that parasitic plant infection would alter the diversity and composition of host plant microbiota, possibly a result of induced physiological and immune host responses, which are known to cause the enrichment of particular microbial taxa (Lebeis *et al.*, 2015; Stringlis *et al.*, 2018; Voges *et al.*, 2019). To address these questions, we investigated the plant-associated bacterial microbiota from stable natural populations of the ivy-parasitizing broomrape *Orobanche hederae*, and infected and uninfected individuals of its host plant, *Hedera* spp. (hereafter *Hedera*).

## Materials and methods

### Study system

*Orobanche hederae* and various species of *Hedera* are naturalized on the University of California, Berkeley (UCB) campus, but are native to Eurasia and North Africa. At UCB, *O. hederae* was first collected in 2000 and has been persistent at several sites for well over 15 years (vouchers at UC and Jepson Herbaria). These plants grow exclusively in near-monocultures of *Hedera algeriensis* or, rarely, *H. helix*. Curiously, the UCB campus is the only record of *O. hederae* outside its native range with the possible exception of a depauperate specimen collected in 1950 in Raleigh, NC but not observed subsequently (Musselman, 1980). Similar to other root holoparasites, *O. hederae* seeds germinate in the presence of a suitable host plant and the seedling immediately attaches to a single host root, where it persists for the remainder of its life (Joel *et al.*, 2013). Organs homologous to the leaves and roots of photosynthetic plants are greatly modified in *O. hederae*: scale-like leaves and short, stout hairless roots composed of large, starch-containing cells surrounding the central vascular bundle (Tate, 1925; Joel *et al.*, 2013).

### Plant and soil sampling

We sampled from four sites across the UCB campus (Fig. 1D). At each site we located one infected and one adjacent but non-contiguous uninfected patch of ivy, assessed by a thorough visual inspection. Infected ivy patches possessed between 20 and 50 live *Orobanche* inflorescences as well as several dozen senesced stalks from previous years, whereas in uninfected patches we were unable to find any living or senesced parasite individuals above- or belowground. Thus, uninfected individuals in our study may represent resistant *Hedera* genotypes or susceptible individuals that have not been confronted with *Orobanche*. Infected and uninfected patches are consistent from year to year (ACS, personal observation from 2013 to 2018). At each infected patch, we carefully excavated flowering *Orobanche* individuals and the connected host plant root, then followed it until we located a connected leaf (Fig. 1B). The above- and belowground organs of the parasite and associated host comprised a single specimen (Fig. 1B). We repeated this process three times in each infected patch, sampling individuals at least 2 m apart. For each uninfected patch, we collected above- and belowground tissue from three ivy individuals at least 2 m apart. Finally, we collected two soil samples (~1 g sampled from 1–10 cm soil depth) from each infected and uninfected patch at each site.

After collection at each site (~2 h), plant samples were immediately brought back to the lab and prepared for DNA extraction of endophytic microbes (Bulgarelli *et al.*, 2012; Lundberg *et al.*, 2012). From infected patches, we harvested samples from parasite leaves and roots (PL and PR, respectively; Fig. 1C), fully expanded ivy leaves (IL), and two ivy root samples (Fig. 1B): infected roots (IIR; i.e. the specific ivy root being parasitized); and uninfected roots (IUR; i.e. roots from the same ivy individual and within 50 cm of the parasite, but not directly being parasitized). From uninfected patches, we harvested ivy leaves and roots (UL and UR, respectively). All samples were carefully selected to represent homologous organs between the parasite and host plant. We used razor blades, rinsed with 70% ethanol between samples, to harvest 500 mg of each sample. From the parasite, we sampled multiple leaves or floral bracts per individual and from the ivy we harvested a small sample from at least two fully expanded leaves per individual.

Samples were placed in 50 ml centrifuge tubes filled with 25 ml of sterile phosphate buffer (Lundberg *et al.*, 2012). We vortexed samples for 15 s and placed them in a new 15 ml tube filled with 5 ml of sterile buffer. After another 15 s vortex, samples were transferred to a new 15 ml tube filled with 5 ml of sterile buffer and vortexed for an additional 15 s. After the final vortex, samples were placed in 1.7 ml microcentrifuge tubes filled with 1 ml of sterile buffer and sonicated (Bioruptor, Diagenode) at low frequency for 5 min (5×: 30 s sonication, 30 s of rest). After sonication, samples were immediately stored overnight at –80 °C and then freeze-dried for 60 h. Due to the physical toughness of the plant tissue, we ground samples with two steel beads (5 mm diameter) in each tube for 2 min prior to DNA extraction (Mini-Beadbeater, Biospec).

### DNA extraction and sequencing

We used extraction kits (DNeasy PowerSoil, Qiagen) to isolate DNA from plant and soil samples, which we then used to amplify the V4 region of the 16S rRNA gene with a dual-index approach (Kozich *et al.*, 2013). To reduce co-amplification of host plant organellar DNA we included peptide nucleic acid (PNA) clamps (Lundberg *et al.*, 2013). Each PCR included: 1.5 μl each of 10 μM forward and reverse indexed primer; 0.17 μl each of 100 μM mitochondrial and plastid PNA; 8.16 μl of H_2_O; 12.5 μl of Kappa 2G Mastermix; and 1 μl of genomic DNA template. We used the following amplification program: 3 min 95 °C; cycle start (15 s 95 °C, 15 s 78 °C, 15 s 50 °C, 15 s 72 °C) cycle end; 5 min 72 °C. Based on band intensity on a 1.0% agarose gel, we determined that plant tissue samples were optimally amplified using 23 cycles and soil samples using 20 cycles. We also included reactions at 23 cycles with sterile H_2_O (negative control), DNA isolated from a pure culture of *Pseudomonas aeruginosa* (positive control), and DNA isolated from a mock community of eight known bacteria. These additional samples were used to ensure that our reagents were not contaminated and that our bioinformatic pipeline yielded the expected number of bacterial taxa in the positive control and mock community. We performed all reactions in triplicate (Applied Biosystems 2720 thermocycler). After pooling triplicate reactions, we fluorometrically quantified the amplified and pooled product from each individual sample (Qubit HS DNA assay, Thermo Fisher Scientific). We then added equal amounts of product from each individual sample to a single tube, purified with AMPure XP beads (Beckman Coulter Inc.), and quantified using the Qubit HS DNA assay. Pooled libraries were sequenced on an Illumina MiSeq using 2× 250 bp paired-end reads at the QB3 Vincent J. Coates Genomics Sequencing Facility at UCB. Individual fastq files for each sample are archived on the NCBI Sequence Read Archive (SRP154488).

### Bioinformatics

After obtaining demultiplexed reads trimmed of primer and index sequences from the Illumina MiSeq, we processed sequencing reads using the R package ‘DADA2’ (v. 1.8.0; Callahan *et al.*, 2016), which infers unique bacterial taxa from amplicon sequence variants (ASVs). Due to poor Phred quality (*Q*) scores, we truncated the length of the forward read to 240 bp and the reverse read to 160 bp. We removed any sequences with ambiguous nucleotide assignment, with any instance of a *Q*-score <2, or with >2 expected errors. After inferring ASVs, we merged forward and reverse sequences and removed chimeras, which resulted in 11.5 million high quality sequences and 21 865 ASVs.

We used the Ribosomal Database Project (RDP) naïve Bayesian classifier (Wang *et al.*, 2007; implemented in DADA2) and the ‘RDP training set 16’ (Cole *et al.*, 2014) to assign taxonomy to individual ASVs. To align ASV sequences and build a maximum likelihood phylogenetic tree, we used the program PASTA with default parameters (v. 1.6.4; Mirarab *et al.*, 2015). Next, we used the R package ‘phyloseq’ to further process our samples (v. 1.24.0; McMurdie and Holmes, 2013). We removed ASVs that were unassigned to a phylum of Eubacteria (268 ASVs removed) or assigned to plastid and mitochondrial lineages (3268 ASVs removed), which left 6.5 million reads distributed across 18 296 ASVs. We identified and removed 33 potential contaminant ASVs, collectively making up 3199 reads or 0.0005% of the total reads in our dataset (Davis *et al.*, 2018). Our final dataset consisted of 102 unique microbiome samples (60 *Hedera* and 24 *Orobanche* samples, 15 soil samples, and three controls), each with on average 63 527 high quality sequences (± 5365; SE).

For downstream analyses of community composition, differential abundance, and network analysis (see below), we applied a prevalence and abundance threshold requiring ASVs to be found in at least five samples (5%) with at least 25 sequences per sample. This yielded 2241 ASVs and accounted for 78% of the total number of sequences in our experimental dataset, and the expected number of ASVs amongst our control samples. For downstream composition analyses, we performed proportional abundance normalization (relative abundance) on this common set of ASVs in each sample (McMurdie and Holmes, 2013). In comparison, we also rarefied our dataset (1000 reads), which yielded ~8291 ASVs and accounted for <2% of the total read count. Both methods yielded qualitatively identical results (data not shown); therefore, we focus our interpretation on the non-rarefied data because they retained a much larger portion of our available data.

### Statistical analyses

#### α- and β-diversity

To test whether α-diversity (the number of taxa within a community) and β-diversity (compositional differences between communities) of leaf and root microbiota varied between *Hedera* and *Orobanche* and between infected and uninfected *Hedera*, we used linear mixed models [LMMs; ‘lmer’ from the R package ‘lme4’ v. 1.1-17 (Bates *et al.*, 2015)]. We calculated α-diversity as ASV richness (R), inverse Simpson’s diversity (D^–1^), and evenness (D^–1^/R). We also calculated phylogenetic diversity (Faith, 1992), which is the sum of the total phylogenetic branch lengths in an assemblage, using the R package ‘picante’ (Kembel *et al.*, 2010). Data for α-diversity indices were log transformed to meet assumptions of normality and homogeneity of variance. We performed principal coordinate analysis (PCoA) using a weighted UniFrac distance matrix of the ASV dataset.

We calculated β-diversity as the individual sample scores along the first three PCoA axes. Plant species (*Hedera* spp. or *O. hederae*), organ type (leaf or root), the species×organ interaction, and total usable reads were treated as fixed effects. Site and the species×site interaction were treated as random effects. Using the model object from ‘lmer’, we tested the significance of fixed effects with type III ANOVA from the R package ‘car’ v. 3.0-0 using the Kenward–Roger degrees of freedom approximation (Fox and Weisburg, 2011). To test the significance of random effects, we used ‘ranova’ from the R package ‘LmerTest’ v. 3.0-1 (Kuznetsova *et al.*, 2017) to perform likelihood ratio tests comparing full and reduced models. Next, we divided the data to include only ivy samples and fit new models to test the effects of infection status on diversity measures. Infection status and usable reads were treated as fixed effects, and site and the site×infection status interaction were treated as random effects.

#### Differential abundance

We used the raw read counts from the common set of ASVs aggregated at each bacterial taxonomic rank to test whether plant species or infection status affected the abundance of individual phyla, classes, orders, families, genera, and ASVs found in leaves and roots. Differential abundance analysis was performed with the R packages ‘DESeq2’ (v. 1.20.0; Love *et al.*, 2014) and ‘ALDEx2’ (v. 1.12.0; Fernandes *et al.*, 2013, 2014). DESeq2 fits negative binomial generalized linear models to variance-stabilized count data (e.g. genes, ASVs, etc.) and estimates the log_2_-fold change in abundance across experimental factors. ALDex2 accounts for the compositional nature of sequencing datasets by performing a scale-invariant transformation on read counts, which are modelled as distributions of posterior probabilities sampled from a Dirichlet distribution. With each method, we tested whether bacterial taxa exhibited differential abundance using specific contrasts: PR versus IIR; PL versus IL; IUR versus IIR; UR versus IIR; UR versus IUR; and UL versus IL. The first two contrasts, PR versus IIR and PL versus IL, test whether bacterial taxa are differentially abundant between *Orobanche* and *Hedera* roots and leaves. The final four contrasts allow us to infer the extent of the effect of *Orobanche* infection on *Hedera* microbiota.

#### Network analysis

To understand how differences between *Orobanche* and *Hedera* and the *Hedera* infection status influenced bacterial community structure, we inferred bacterial co-association networks (Layeghifard *et al.*, 2017) for each of the leaf and root community types occurring in *Orobanche* and *Hedera* [i.e. PL, PR, UL, UR, IL, IR, and IIR (*n*=12 for each type)]. Network inference yields a set of bacterial ASVs (nodes) connected by edges, which represent significant co-associations (either positive or negative co-associations occurring across samples). The pattern of nodes and edges in a given community can be characterized by summary statistics and compared with other networks in ways that we describe in more detail below.

To infer co-association networks, we used two methods that are designed to be robust to the compositional and sparse nature of microbiome datasets, SPIEC-EASI (Kurtz *et al.*, 2015) and SparCC (Friedman and Alm, 2012). For SPIEC-EASI network inference, we used the function ‘spiec.easi’ from the R package ‘SpiecEasi’ v. 0.1.4 (method=‘mb’, min.lambda.ratio=0.002, nlambda=100, and rep.num=30). We implemented SparCC using the functions ‘sparcc’, ‘sparccboot’, and ‘pval.sparccboot’ from the SpiecEasi package. We used default parameters for the main SparCC algorithm and calculated pseudo-*P*-values from 100 bootstrapped datasets. We retained associations between ASVs that had an absolute correlation >0.6 and a pseudo-*P*-value <0.05 (Friedman and Alm, 2012). To reduce the bias of anomalous ASVs found at particular sampling sites, we included only ASVs found with at least 10 reads in 50% of samples (Berry and Widder, 2014). We applied this threshold for each community type separately because some ASVs were unique to single community types (e.g. Supplementary Fig. S3 and Supplementary Table S4 at *JXB* online) and thus would have been filtered out had the threshold been applied to the entire dataset. This resulted in seven ASV subsets representing a fraction of the total sequenced reads for each community type (number of ASVs/fraction of total: IIR, 229/63%; IUR, 285/61%; UR, 289/60%; PR, 113/51%; IL, 11/50%; UL, 10/54%; and PL, 23/56%).

For each network, we calculated ASV-level and whole-network properties thought to be related to individual and community function (Röttjers and Faust, 2018) and compared them using the R package ‘igraph’ v. 1.2.2 (Csardi and Nepousz, 2006). First, we calculated two measures of individual ASV centrality: degree and betweenness centrality. Degree is the number of edges connected to an ASV, and betweenness centrality measures the extent to which an ASV lies on edges connecting other ASVs (Freeman, 1978). ASVs with a high degree number have numerous co-associations with other network members and may be indicative of hub species (Agler *et al.*, 2016), while ASVs with high betweenness centrality may be mediating interactions between other network members (Röttjers and Faust, 2018). For each network, we calculated the mean of 50 ASV centrality measures sampled with replacement from the observed values. After repeating this process 10 000 times, we compared the distributions of mean node centrality among networks using a series of Kolmogorov–Smirnov tests.

At the whole-network level, we calculated edge density and network betweenness centrality. Edge density measures the observed proportion of all possible co-associations among ASVs. Low edge density may indicate that species assemblages lack persistent interactions. Network betweenness centrality measures the evenness of betweenness centrality among network members (Freeman, 1978). Values of network betweenness centrality close to zero indicate that all ASVs are equally central, and values close to one indicate high variance in the individual betweenness centrality among ASVs.

#### Procrustes tests

To test whether *Orobanche* microbiota track those of their ivy host, we performed a series of Procrustes analyses (Peres-Neto and Jackson, 2001). We used Procrustes analysis to match corresponding sample scores between two community types [i.e. PL, PR, UL, UR, IL, IR, and IIR (*n*=12 for each type)] along the first two principal coordinate axes (PCoA 1 and PCoA 2) in a PCoA performed on the weighted UniFrac distances among samples. The congruence between two bacterial communities is given by the Procrustes correlation-like statistic (*t*_0_), which ranges from 0 (complete discordance) to 1 (perfect congruence). High congruence between two community types (e.g. IIR and PR) indicates that compositional shifts in one community are closely matched by parallel compositional shifts in the other community across samples (Supplementary Fig. S7). To understand how individual bacterial taxa may be contributing to the congruence or discordance between parasite and host microbiota, we used a leave-one-out approach (Wang *et al.*, 2012). We removed all bacterial ASVs from the parasite dataset classified to a given bacterial phylum, recalculated weighted UniFrac distances among all samples, obtained sample scores from a new PCoA, and recalculated *t*_0_. The effect of excluding a particular bacterial clade on the fit between host and parasite microbiota is given by Δ*t*=(*t*_excluded_*–t*_0_). After repeating this for each bacterial phylum, we iterated the leave-one-out approach across bacterial orders within phyla whose exclusion lead to large Δ*t*. We performed the entire leave-one-out approach on root and leaf bacterial communities separately (PR versus IIR; PL versus IIR). We used the function ‘protest’ with 999 permutations from the R package ‘vegan’ v. 2.5-2 (Oksanen *et al.*, 2018), which calculates *t*_0_, and performs the permutation test.

All data and R codes used in the analyses are available on the Dryad Digital Repository (https://doi.org/10.5061/dryad.7wm37pvnk).

## Results

### *Orobanche* root microbiota are less diverse, compositionally dissimilar, and display far fewer co-associations than *Hedera*

**Fig. 2.**
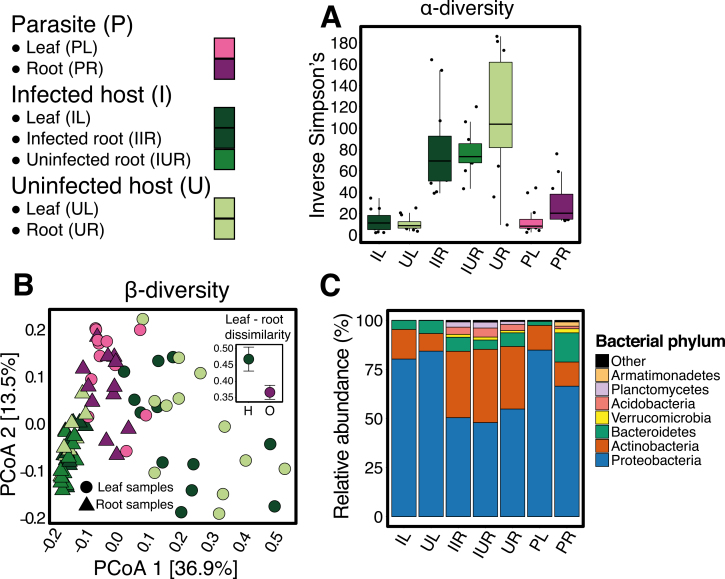
Diversity and composition of the bacterial communities inhabiting *Orobanche* and *Hedera*. See Supplementary Tables S2 and S3 for test statistics; *P*<0.001 unless otherwise indicated. (A) Inverse Simpson’s diversity of each tissue/species/infection status combination (*n*=12). Roots exhibited nearly 4-fold higher diversity than leaves. Parasites had reduced root, but not leaf diversity relative to hosts. *Hedera* infection status had no effect on leaf or root diversity. (B) Principal coordinates analysis of the weighted UniFrac dissimilarity among bacterial communities showing a distinct microbiota inhabiting leaves and roots (point shape) and between plant spieces (point colors). Inset: a significant species×organ type interaction reflects larger compositional differences found between leaf and root microbiota inhabiting *Hedera* than those inhabiting *Orobanche* (*P*=0.01). (C) The relative abundance of the major bacterial phyla found across leaves and roots of both plant species (*n*=12 for each community type).

*Orobanche* roots harbored reduced species (ASV) richness of bacteria relative to *Hedera*, although leaves of the two species had similar richness (Fig. 2A; Supplementary Fig. S1; Supplementary Table S2). In both species, root communities were compositionally distinct from the respective leaf communities (Fig. 2B, C; Supplementary Fig. S2; Supplementary Table S3: *F*_1,22_=64.04, *P*<0.001), and displayed greater richness but reduced evenness (Fig. 2A; Supplementary Fig. S1; Supplementary Table S2). However, the highest bacterial richness was found in the soil samples collected from each site (Supplementary Fig. S1).

When considering the identity of individual ASVs, leaf and root communities of *Orobanche* were compositionally distinct from those of *Hedera* samples (Fig. 2B, C; Supplementary Fig. S2; Supplementary Table S3: *F*_1,12_=51.32, *P*<0.001), with a total of 246 unique ASVs found only in *Orobanche* (Supplementary Table S4). However, the bacterial communities inhabiting leaves and roots of *Orobanche* exhibited greater compositional similarity with each other than those inhabiting leaves and roots of *Hedera* (Fig. 2B inset; paired *t*-test: *t*= –2.52, *P*=0.01). In other words, the leaf and root communities of *Orobacnhe* were more homogeneous than those of *Hedera*. Despite a lower richness overall, *Orobanche* leaves and roots shared a greater number and proportion of ASVs than *Hedera* leaf and root communities. Fourteen percent of the total ASVs found in *Orobanche* leaves and roots were shared between these communities, while only 2% were shared between *Hedera* leaves and roots (Supplementary Fig. S3C)

**Fig. 3.**
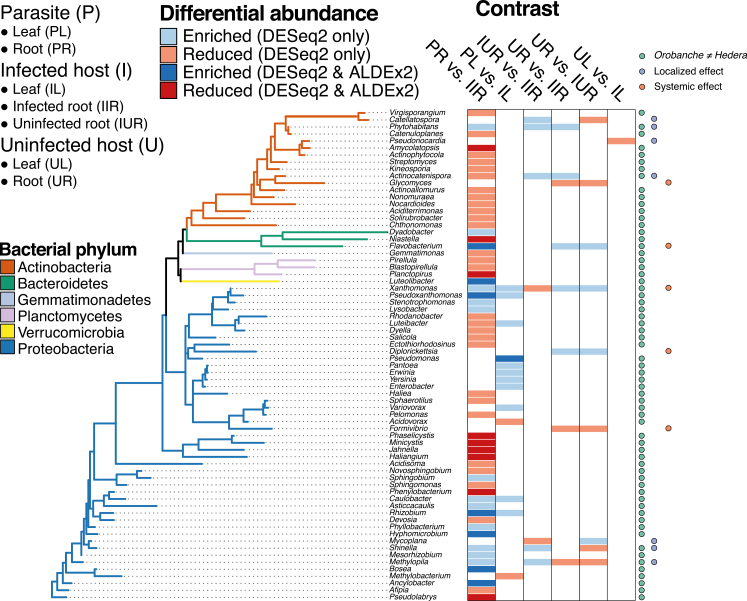
Differential abundance of bacterial genera (grouped by phylum) across five plant species, tissue, or infection status contrasts (*n*=12, abbreviations follow Figs 1 and 2). For example, ‘PR versus IIR’ tested whether bacterial families in *Orobanche* roots (PR) exhibited enriched or reduced abundance relative to infected *Hedera* roots (IIR). We used two analytical methods to test for differential abundance, DESeq2 and ALDEx2; significant differences in results revealed by both methods are displayed in darker shades and the results unique to DESeq2 in lighter shades. Colored circles indicate bacterial genera enrichment patterns consistent with differences between *Orobanche* and *Hedera* (green), or localized (purple) or systemic (orange) effects of infection status on bacteria inhabiting *Hedera.* Results for other taxonomic ranks are shown in Supplementary Fig. S4 and Supplementary Table S5.

Individual bacterial taxa also varied in their relative abundance. Many bacterial taxa across all taxonomic ranks were differentially abundant between *Orobanche* and *Hedera* roots and to a lesser extent leaves (illustrated at the genus level in Fig. 3; other ranks in Supplementary Fig. S4 and Supplementary Table S5). Relative to *Hedera*, *Orobanche* roots exhibited an increased abundance of the phyla Armatimonadetes, Bacteroidetes, and Proteobacteria, whereas the Planctomycetes were conspicuously absent (Fig. 2C; Supplementary Table S5). The families Cytophagaceae, Flavobacteriaceae, Phyllobacteriaceae, Rhizobiaceae, and Verrucomicrobiaceae were all enriched in *Orobanche* roots relative to *Hedera* roots, while Haliangiaceae, Micromonosporaceae, Phaselicystidaceae, Polyangiaceae, Rhizomicrobium, and Streptomycetaceae were depleted (Supplementary Table S5). Relative to *Hedera* leaves, *Orobanche* leaves had a higher abundance of Acidobacteria, Proteobacteria, and Verrucomicrobia (Fig. 2C; Supplementary Table S5), including enrichment of members of the well-known plant-colonizing bacterial families Enterobacteriaceae, Pseudomonadaceae, and Rhizobiaceae.

**Fig. 4.**
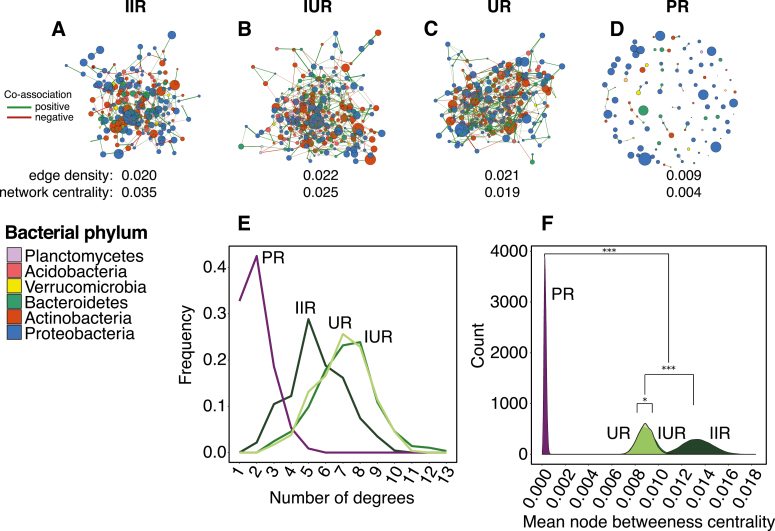
(A–D) *Orobanche* and *Hedera* root bacterial networks inferred by SPIEC-EASI large differences in the edge density and betweenness centrality between *Hedera* and *Orobanche*, but not across infected and uninfected *Hedera* roots (see Supplementary Table S6) (A) *Hedera* roots that are directly being parasitized by *Orobanche* (IIR), (B) uninfected roots of infected *Hedera* plants (IUR), (C) roots of uninfected *Hedera*, and (D) *Orobanche* roots. Node color and size represent bacterial phylum classification and abundance (centered log-ratio transformed) respectively. Color and width represent the sign (green, positive association; red, negative association) and strength of co-association, respectively. The network properties can be summarized by (E) degree distribution (number of associations per node) among community types and (F) mean node betweenness centrality, which varied significantly among the root bacterial networks of *Hedera* and *Orobanche*, as well as infected and uninfected *Hedera* roots. We tested significance using a series of Kolmogorov–Smirnov tests on the distributions of mean node-level betweenness centrality estimated from 50 nodes sampled with replacement 10 000 times (*P<0.05; ***P<0.001)

In addition to differences in diversity and composition, the structure of root microbiota as measured by bacterial network attributes differed between *Orobanche*, infected *Hedera*, and uninfected *Hedera* (Fig. 4; Supplementary Fig. S5; Supplementary Table S6). Compared with *Hedera*, the root bacterial network of *Orobanche* displayed a near absence of co-associations among ASVs (Fig. 4A–D). This absence was reflected in the measure of mean ASV centrality, which was ~10-fold lower in *Orobanche* versus *Hedera* root bacterial networks (Fig. 4F, Kolmogorov–Smirnov test, *D*=1, *P*<0.001; results are qualitatively similar for degree number). The lack of co-associations in the *Orobanche* root bacterial network is also evident from the diminished number of associations per bacterial ASV (i.e. degree distribution) in the *Orobanche* network (Fig. 4E). Additionally, the edge density and network betweenness centrality of the *Orobanche* root bacterial network were substantially reduced relative to *Hedera* (Fig. 4A–D), indicating that the reduced number of co-associations in the *Orobanche* root bacterial network is an attribute shared among all constituent ASVs. In contrast to plant roots, we found few significant associations between members of leaf bacterial communities (Supplementary Fig. S6; Supplementary Table S6).

### *Orobanche* leaf and root microbiota exhibit congruency with *Hedera* roots

**Fig. 5.**
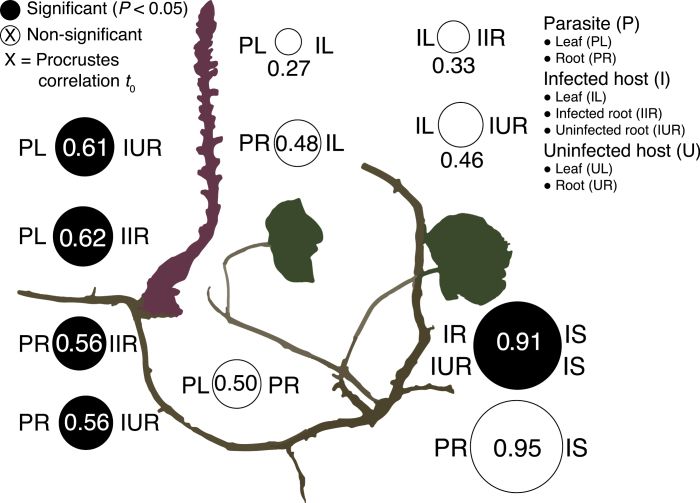
Congruence in compositional change across *Hedera* and *Orobanche* leaves and roots. Circle size is proportional to the Procrustes correlation between PCoA ordinations of weighted UniFrac dissimilarity. Filled circles are significant at *P*<0.05). For example, compositional change of *Orobanche* leaf bacterial communities (PL) across replicates was not correlated (*t*_0_=0.5, *P*>0.05, *n*=12) with compositional change in *Orobanche* roots (PR). Instead turnover among PL communities was positively correlated with compositional change among both infected (IIR: *t*_0_=0.62, *P*<0.05, *n*=12) and uninfected (IUR: *t*_0_=0.61, *P*<0.05, *n*=12) roots of infected hosts. See Supplementary Table S7 for additional comparisons.

Individual *Orobanche* leaf and root communities significantly resembled the infected (*Orobanche* leaf: *t*_0_=0.62, *P*=0.02; *Orobanche* root: *t*_0_=0.56, *P*=0.03) and uninfected roots (*Orobanche* leaf: *t*_0_=0.61, *P*=0.02; *Orobanche* root: *t*_0_=0.56, *P*=0.02) of their *Hedera* hosts (Fig. 5; Supplementary Table S7). Leaf and root communities were not congruent within a given species (*Orobanche*: *t*_0_=0.50, *P*=0.11; *Hedera*: *t*_0_=0.33, *P*=0.53). *Hedera* root communities were congruent with soil bacterial communities in infected but not uninfected patches (infected patches: *t*_0_=0.91, *P*=0.04; uninfected: *t*_0_=0.83, *P*=0.33). *Orobanche* roots displayed high but non-significant congruence to soil communities in infected patches (*t*_0_=0.95, *P*=0.08).

Our leave-one-out approach to the Procrustes tests revealed that a subset of bacterial taxa contribute strongly to either the congruence or discordance between *Orobanche* and *Hedera* leaf and root communities (Supplementary Figs S7–S10; Supplementary Tables S8, S9). In root communities, excluding the Burkholderiales (Proteobacteria) led to reduced congruence between *Orobanche* and *Hedera* root microbiota, as shown by a large decrease in the Procrustes goodness of fit (Δ*t*_0_= –0.133). In contrast, excluding the Actinomycetales (Actinobacteria) and Flavobacteriales (Bacteroidetes) led to large increases in the Procrustes goodness of fit (Actinomycetales, Δ*t*_0_=0.043; Flavobacteriales, Δ*t*_0_=0.032), which indicates that these taxa contribute to dissimilarity between *Orobanche* and *Hedera* root microbiota. The relative abundance of Burkholderiales, but not Actinomycetales, was strongly correlated in *Orobanche* and *Hedera* roots, further supporting that the Burkholderiales are a strong contributor to congruence between root communities of parasite and host (Supplementary Fig. S9). The fit between *Orobanche* leaf and *Hedera* root communities worsened after excluding Pseudomonadales (Δ*t*_0_= –0.112) and Rhizobiales (Δ*t*_0_= –0.012), whereas the fit increased after excluding Enterobacteriales (Δ*t*_0_=0.032) and Sphingomonadales (Δ*t*_0_=0.030), all bacterial orders from the Proteobacteria.

### Infection status has modest effects on the abundance of individual bacterial taxa and network attributes in *Hedera* bacterial communities

In contrast to our predictions, infection status had no effect on the overall diversity or composition of *Hedera* leaf and root bacterial communities (Fig. 2A–C; Supplementary Tables S2, S3), though we found a significant interaction between root infection status and sampling site (Supplementary Table S3; χ ^2^=9.12, *P*=0.003. Although several ASVs were unique to infected leaves and roots (Supplementary Table S4), few bacterial taxa were affected by infection status, and these findings appeared to be sensitive to the analysis method (Fig. 3; Supplementary Fig. S4; Supplementary Table S5). Considering only DESeq2 results, which identified more differentially abundant taxa than ALDEx2, a number of taxa did exhibit differential abundance in infected *Hedera* roots and leaves consistent with either localized or systemic effects of parasitic plant infection on host plant microbiota (Fig. 3: localized effects, purple circles; systemic effects, orange circles). Moreover, our network analyses revealed that infected *Hedera* root bacterial communities had higher mean ASV and network betweenness centrality than uninfected roots from both infected and uninfected individuals (Fig. 4F; Kolmogorov–Smirnov test: *D*=0.45, *P*<0.001). Thus, *Orobanche* infection leads to an increase in the mean but also increased variance in centrality, indicating that this may be driven by changes in particular ASVs in *Hedera* roots.

## Discussion

### The bacterial microbiota of a root holoparasite

The lower bacterial diversity and simpler co-association networks we found in *Orobanche* roots parallel their reduced morphological and anatomical structure (Figs 1B, C) (Tate, 1925), and are perhaps due to the availability of fewer microbial niches relative to autotrophic plants. Additionally, *Orobanche* and *Hedera* differed in the composition of both leaf and root bacteria (Fig. 2B, C). Variation in functional traits (e.g. leaf and root mass per area, or leaf nitrogen content) and ecological strategies among plants are thought to contribute to differences in leaf and root microbiota among plant species (Kembel *et al.*, 2014; Laforest-Lapointe *et al.*, 2016; Fitzpatrick *et al.*, 2018). Our results support this paradigm for bacterial composition and root bacterial diversity, but foliar bacterial diversity appears less sensitive to a shift to heterotrophy in their host than root-associated bacteria (Fig 2B; Supplementary Fig. S4; Supplementary Table S5).

Like other ecological communities, interactions among microbial species are important determinants of the overall composition and function of plant microbiota (Durán *et al.*, 2018; Carlström *et al.*, 2019; Finkel *et al.*, 2019b, Preprint). Remarkably, we found a near absence of microbial co-associations in *Orobanche* roots, in contrast to *Hedera* roots where we robustly identified numerous co-associations (Fig. 4). Shi *et al.* (2016) proposed that the increased complexity of microbial networks found in rhizosphere versus bulk soil could be due to increased interactions including microbial cross-feeding, competition, and other forms of antagonism. However, niche differences can also result in significant negative and positive co-associations among microbial taxa as environmental variation across habitats, in this case host roots or shoots, drives species co-occurrence (Zhou *et al.*, 2011; Shi *et al.*, 2016; Freilich *et al.*, 2018). In the context of plant roots, simpler bacterial networks could be the result of fewer persistent microbial interactions and/or a reduced number of available niches across sampled plants. This is not to say that microbial interactions or niche differences are absent but rather that they are inconsistently acting as drivers of bacterial community composition across *Orobanche* roots. Instead, our results indicate a greater role for stochastic processes in *Orobanche* root bacterial community dynamics. This remains to be tested experimentally, but is plausible given that the function of nutrient uptake has been supplanted by the haustorium, leaving the *Orobanche* roots more or less vestigial (Joel *et al.*, 2013).

The functional consequences of the patterns of bacterial diversity and enrichment associated with heterotrophic versus autotrophic plants shown here remain unknown and require further study using a range of plant parasites and hosts. The Orobanchaceae include the full spectrum of trophic life histories, including free-living autotrophs, and facultative and obligate parasites such as *O. hederae*. This presents a unique opportunity to test how variation in the degree of parasitism, and thus heterotrophy, shapes microbial dynamics in both hosts and parasites. Nonetheless, based on the reduced diversity and simpler network structure in this host–parasite system, we propose that the concept of ‘parasitic reduction syndrome’, referring to the long-recognized morphological, functional, and genetic reduction accompanying the transition to a heterotrophic lifestyle (Tate, 1925; Kuijt, 1969; Colwell, 1994), be extended to include a reduction in microbiome diversity and structure as well.

### Assembly of the parasitic plant microbiota

In this study, *Orobanche* leaf and root bacterial communities tracked the composition of root bacterial communities observed in infected *Hedera* across replicate sites (Fig. 5; Supplementary Table S6). Studies in other systems provide additional evidence that the assembly of parasitic plant microbiota is likely to be shaped by factors associated with host plants (Sheng-Liang *et al.*, 2014; Kruh *et al.*, 2017; Cui *et al.*, 2018). Importantly, we found strong congruency between surrounding soil bacterial composition and the root communities of *Hedera*, but not *Orobanche*, indicating that soil environmental features are not solely driving corresponding shifts in parasite and host root microbiota (Fig. 5; Supplementary Table S6). Holoparasitic plants such as *Orobanche* obtain their requisite energy, nutrients, and water from their hosts by way of haustoria, which also allow symplastic and apoplastic transfer of nucleic acids, proteins, and microorganisms (LeBlanc *et al.*, 2012; Yoshida *et al.*, 2016, Spallek *et al.*, 2017. This haustorial transfer of microorganisms and plant-derived molecules that may act to structure microbiota occurring in both *Orobanche* roots and leaves, could drive this congruence (Kruh *et al.*, 2017). For example, *O. hederae* can sequester antimicrobial polyacetylenes from ivy hosts (Avato *et al.*, 1997; Sareendenchai and Zidorn, 2008). *Orobanche* sequestration coupled with host plant variation in the identity or abundance of such molecules would lead to congruence in the composition of associated microbiota between parasite and host.

Particular bacterial taxa contributed most strongly to our observed congruence (e.g. Burkholderiales) or discordance (e.g. Actinomycetales) between *Orobanche* and *Hedera* microbiota (Supplementary Figs S7, S8; Supplementary Tables S8, S9), indicating that the abundance of these bacteria is tightly linked or decoupled, respectively, between parasitic plants and their host. Moreover, that different bacterial taxa contribute to the congruence of either *Orobanche* leaf and *Hedera* root communities or *Orobanche* root and *Hedera* root communities (Supplementary Figs S7, S8; Supplementary Tables S8, S9) lends support to our previous finding that the mechanisms structuring parasite leaf and root communities are not entirely overlapping. Among the four bacterial genera that were enriched in *Orobanche* leaves and roots relative to *Hedera* (Fig. 3), only *Rhizobium* has been studied in relation to parasitic plant function. Researchers have found both positive (Morozov *et al.*, 2000) and negative (Mabrouk *et al.* 2007) effects of rhizobia inoculations on the germination and success of broomrapes parasitizing legume hosts, mediated in part by rhizobia-induced host plant metabolites and growth (Mabrouk *et al.* 2007). Why *Rhizobium* was enriched in *Orobanche* roots and leaves in our study is unclear.

The leaf and root communities inhabiting *Orobanche* were compositionally more similar than leaf and root communities inhabiting *Hedera* (Fig. 2B inset). Moreover, the reduced complexity of the *Orobanche* root bacterial network further indicates a diminished role for microbe–microbe interactions and niche-based processes in the assembly of the parasitic plant microbiome (Fig. 4; Supplementary Figs S5, S6; Supplementary Table S6).Two non-exclusive explanations for this are (i) increased overlap in the microbial habitats of *Orobanche* leaves and roots versus *Hedera*, perhaps a result of increased functional homogeneity of holoparasitic plant organs; or (ii) a larger role for stochastic processes governing the assembly of *Orobanche* microbiota. More broadly, these patterns of *Orobanche* bacterial diversity and community assembly exhibit intriguing parallels with the microbiota of eukaryotic parasites of animal hosts (e.g. Husnik, 2018) and suggest that general microbial dynamics may exist in host–parasite systems across plant and animal kingdoms (Dheilly, 2014; Dheilly *et al.*, 2015).

### The effect of infection status on host plant microbiota

Infection by a parasitic plant can induce host plant morphological, physiological, molecular, and transcriptional responses (Westwood *et al.*, 1998; Castillejo *et al.*, 2009; Hiraoka *et al.*, 2009; Hegenauer *et al.*, 2016). In response to infection by the root holoparasite *Orobanche cernua*, sunflowers synthesize coumarins (Serghini *et al.*, 2001), compounds that are known to inhibit fungal pathogens and also reshape the root microbiome due to antimicrobial effects (Stringlis *et al.*, 2018, 2019). In our study, several bacterial genera exhibited differential abundance consistent with either localized or systemic effects of parasitic plant infection on host microbiota (Fig. 3). *Phytohabitans* was reduced in only directly infected roots, while *Flavobacterium* was reduced in both infected and uninfected roots from infected individuals, indicative of systemic effects of infection on *Hedera* root microbiota. Kruh *et al.* (2017) found that *Flavobacterium* was enriched in the post-attachment but pre-inflorescence stage of the parasitic *Phelipanche aegyptiaca* during infection of tomato plants. In spite of these shifts in taxon abundance, we found that infection status had no effect on the overall diversity or composition of *Hedera* microbiota. In contrast, Kruh *et al.* (2017) found that the microbiota of tomato roots parasitized by *P. aegyptiaca* in a greenhouse setting exhibited compositional similarity to that of their parasite when infected, but also were distinct from the roots of uninfected hosts. However, we found that infected *Hedera* root bacterial networks exhibited elevated network- and ASV-level betweenness centrality relative to uninfected *Hedera* roots (Fig. 4), suggesting that particular ASVs become increasingly co-associated with others in infected roots. Alternatively, under infection, host roots may promote the development of novel microbial niches, which could perturb the co-associations among bacterial taxa, resulting in an increase in the co-occurrence of a small number of taxa (Shi *et al.*, 2016). Recent work demonstrates that root-associated microbiota of host plants may play an important role in mitigating the negative effects of parasitic plant infection (Sui *et al.*, 2018; Masteling *et al.*, 2019). To mechanistically link infection status and microbiota, future work should characterize the effect of microbial inoculations across resistant and susceptible genotypes during experimentally controlled parasitic plant infections (e.g. Castillejo *et al.*, 2009; Castrillo *et al.*, 2017).

## Supplementary data

Supplementary data are available at *JXB* online.

Fig. S1. Measures of α-diversity across plant and soil samples.

Fig. S2. PCoA ordinations and scree plots for different measures of community dissimilarity.

Fig. S3. Shared ASVs among leaf and root communities.

Fig. S4. The proportion of bacterial taxa affected by plant species and *Hedera* infection status.

Fig. S5. Root bacterial networks inferred using SparCC.

Fig. S6. Leaf bacterial networks inferred using SPIEC-EASI.

Fig. S7. Procrustes residuals and bacterial community dendrograms.

Fig. S8. Leave-one-out Procrustes analysis comparing *Orobanche* and *Hedera* root communities.

Fig. S9. Relative abundance of bacterial taxa that contribute to the congruence and discordance between *Orobanche* and *Hedera* root communities.

Fig. S10. Leave-one-out Procrustes analysis comparing *Orobanche* leaf and *Hedera* root communities.

Fig. S11. Principle coordinate analysis of soil bacterial communities.

Table S1. Genome sizes and sampling locations for each *Hedera* spp. individual.

Table S2. Linear mixed effect model results for the analysis of α-diversity.

Table S3. Linear mixed effect model results for the analysis of β-diversity.

Table S4. Bacterial ASVs unique to *Orobanche* and *Hedera* leaves and roots.

Table S5. Full differential abundance testing results.

Table S6. Network attributes of bacterial communities in *Orobanche* and *Hedera.*

Table S7. Results from Procrustes analyses.

Table S8. Results from the leave-one-out Procrustes analysis at the level of bacterial phyllum comparing *Orobanche* and *Hedera* root communities.

Table S9. Results from the leave-one-out Procrustes analysis at the level of bacterial order comparing *Orobanche* and *Hedera* root communities.

erz572_suppl_Supplementary_Figures_S1-S11_and_Tables_S1-S3_S6-S9

erz572_suppl_Supplementary_Tables_S4-S5

## Data availability

The ASV table, bacterial 16S tree, ASV taxonomy, and R script are available at Dryad Digital Repository: https://doi.org/10.5061/dryad.7wm37pvnk (Fitzpatrick and Schneider, 2020)

Sequences are available at NCBI Sequence Read Archive (SRP154488).

Plant vouchers are deposited at the United States National Arboretum Herbarium (NA). Vouchers labeled ‘Schneider & Fitzpatrick’ followed by the collection number are indicated in Table S1.
