# Supplementary material for: Unique bacterial assembly, composition, and interactions in a parasitic plant and its host
Source: J Exp Bot. 2020 Jan 6;71(6):2198–209. doi: 10.1093/jxb/erz572 (PMC7094075; doi:10.1093/jxb/erz572)
Supplement: erz572_suppl_Supplementary_Figures_S1-S11_and_Tables_S1-S3_S6-S9 [file erz572_suppl_supplementary_figures_s1-s11_and_tables_s1-s3_s6-s9.pdf]

Supplementary Data

Article title: Unique bacterial assembly, composition, and interactions in a parasitic plant and its host.

Fig. S1 Measures of α-diversity across plant and soil samples.
Fig. S2 Principle coordinate analyses and corresponding scree plots associated with different measures of community dissimilarity.
Fig. S3 Shared ASVs among leaf and root communities.
Fig. S4 The proportion of bacterial taxa affected by plant species and *Hedera* infection status.
Fig. S5 Root bacterial networks inferred using SparCC.
Fig. S6 Leaf bacterial networks inferred using SPIEC-EASI.
Fig. S7 Procrustes residuals and bacterial community dendrograms.
Fig. S8 Leave-one-out Procrustes analysis comparing *Orobanche* and *Hedera* root communities.
Fig. S9 The relative abundance of bacterial taxa that contribute to the congruence and discordance between *Orobanche* and *Hedera* root communities.
Fig. S10 Leave-one-out Procrustes analysis comparing *Orobanche* leaf and *Hedera* root communities.
Fig. S11 Principle coordinate analysis of soil bacterial communities.
Table S1 Genome sizes and sampling locations for each *Hedera* spp. individual.
Table S2 Linear mixed effect model results for the analysis of α-diversity.
Table S3 Linear mixed effect model results for the analysis of β-diversity.
Table S4 Bacterial ASVs unique to *Orobanche* and *Hedera* leaves and roots.
Table S5 Full differential abundance testing results.
Table S6 Network attributes of bacterial communities in *Orobanche* and *Hedera.*
Table S7 Results from Procrustes analyses.
Table S8 Results from the leave-one-out Procrustes analysis comparing *Orobanche* and *Hedera* root communities.
Table S9 Results from the leave-one-out Procrustes analysis comparing *Orobanche* and *Hedera* root communities.

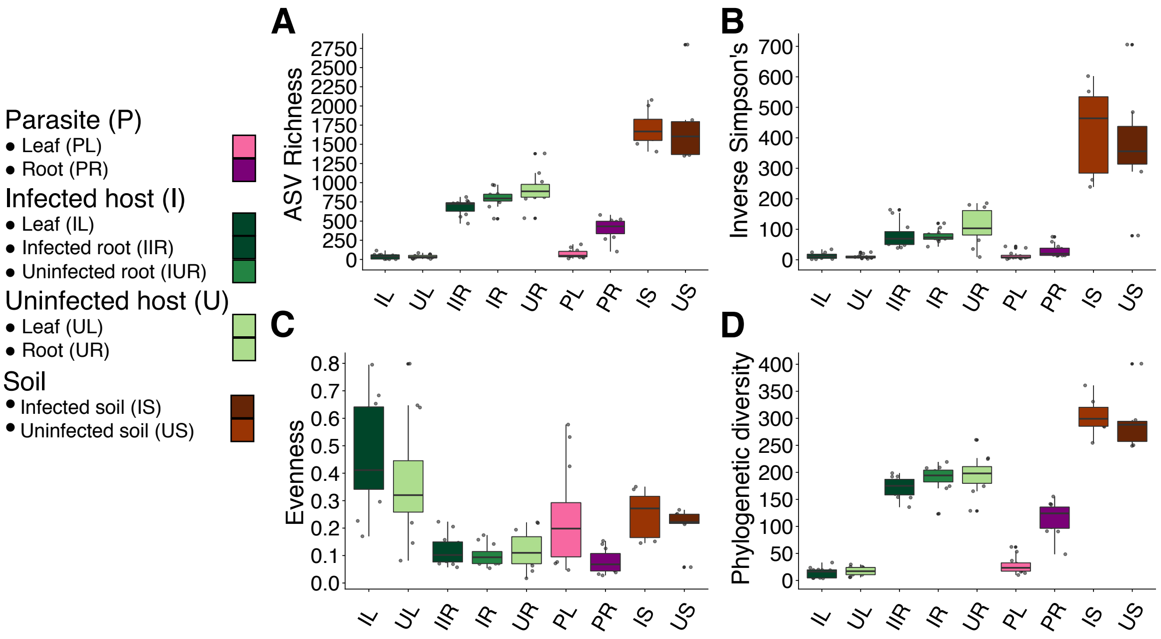

Figure S1 Measures of (A) ASV richness, (B) Inverse Simpson's, (C) evenness, and (D) phylogenetic diversity across leaves and roots from *Hedera* and *Orobanche* and soil samples. Evenness was calculated as inverse Simpson's diversity/ASV richness and phylogenetic diversity was calculated as the sum of the total phylogenetic branch length for a given sample. For results from statistical analyses see Table S2.

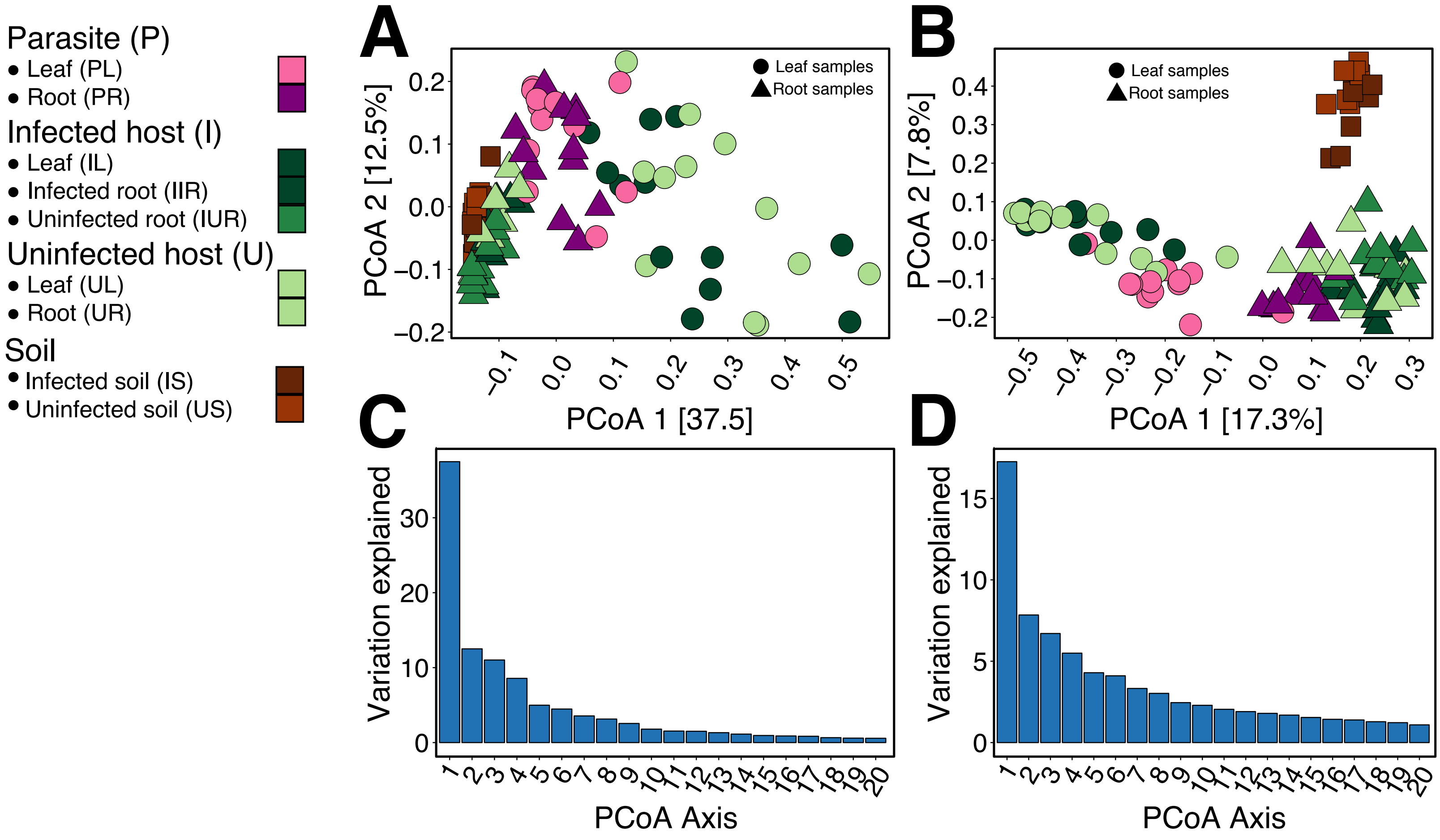

Figure S2 Principle coordinate analyses and corresponding scree plots using (A and C) weighted UniFrac dissimilarity and (B and D) Bray-Curtis dissimilarity. Results were qualitatively similar between measures of dissimilarity.

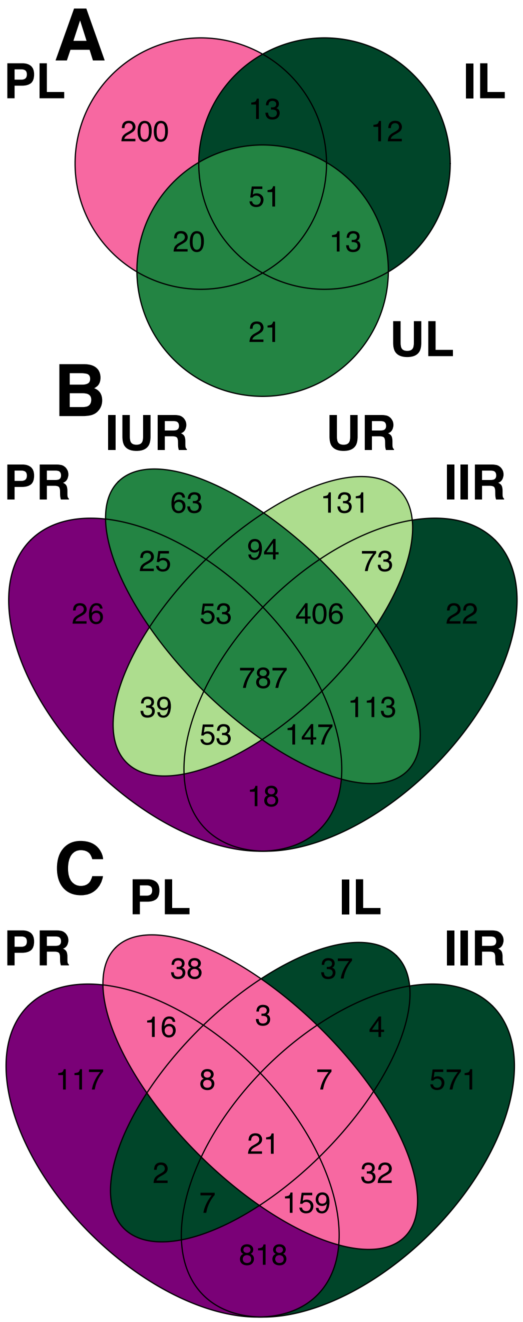

Figure S3 Venn diagrams displaying the number of shared ASVs among (A) leaf communities, (B) root communities, and (C) leaf and root communities.

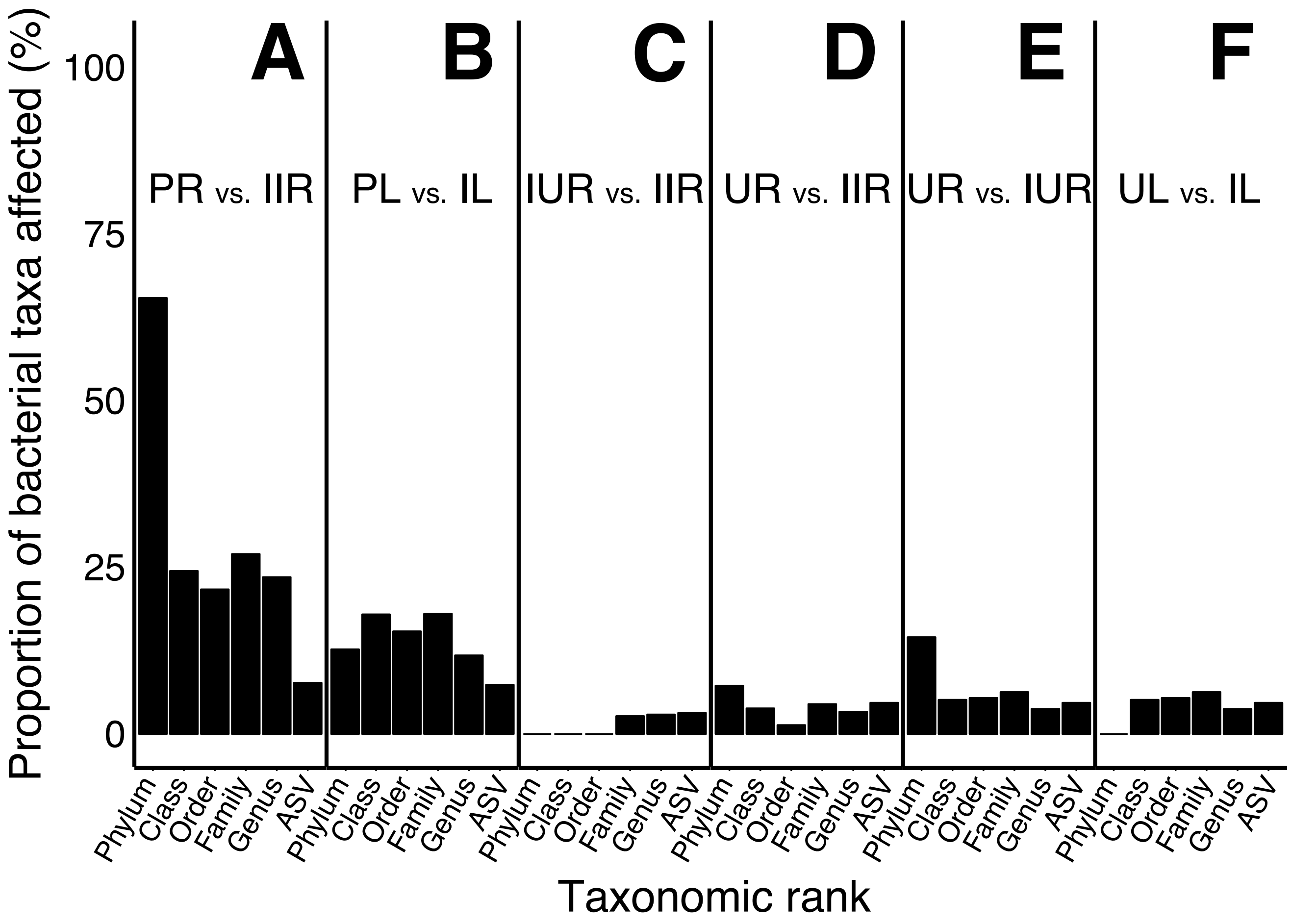

Figure S4 The proportion of bacterial taxa exhibiting significant differential abundance between *Orobanche* and *Hedera* (A: roots; and B: leaves), or *Hedera* infection status (C, D, E and F) using either DESeq2 or ALDEx2 (see Materials and Methods).

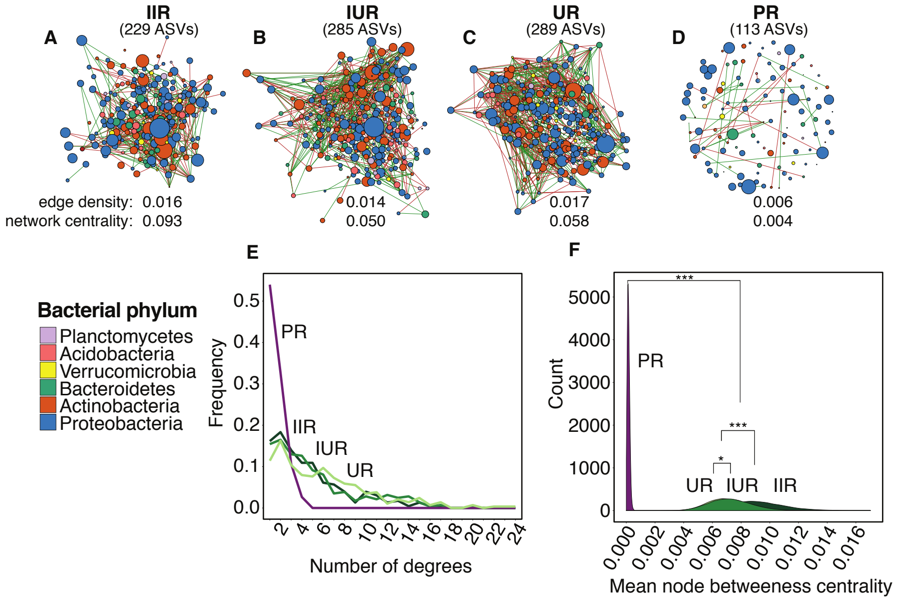

Figure S5 Root bacterial networks of *Orobanche* and *Hedera* inferred using SparCC. We inferred the bacterial network of (A) infected roots of infected *Hedera,* (B) uninfected roots of infected *Hedera*, (C) roots of uninfected *Hedera*, and (D) *Orobanche.* Node colour and size represent bacterial phylum classification and abundance (centered log-ratio transformed), respectively. Edge colour and width represent sign (green = positive association, red = negative association), and strength of co-association, respectively. At the whole network-level, we found large differences in the edge density and betweenness centrality between *Hedera* and *Orobanche*, but not across infected and uninfected *Hedera* roots, as reflected in the (E) degree distribution (number of associations per node) between community types. (F) At the level of individual nodes we found large significant differences in mean betweeness centrality between taxa within the root bacterial networks of *Hedera* and *Orobanche*, as well as infected and uninfected *Hedera* roots. We tested significance using a series of Kolmogorov-Smirnov tests on the distributions of mean node-level betweeness centrality estimated from 10,000 samples with replacement of 50 nodes (see Materials and Methods).

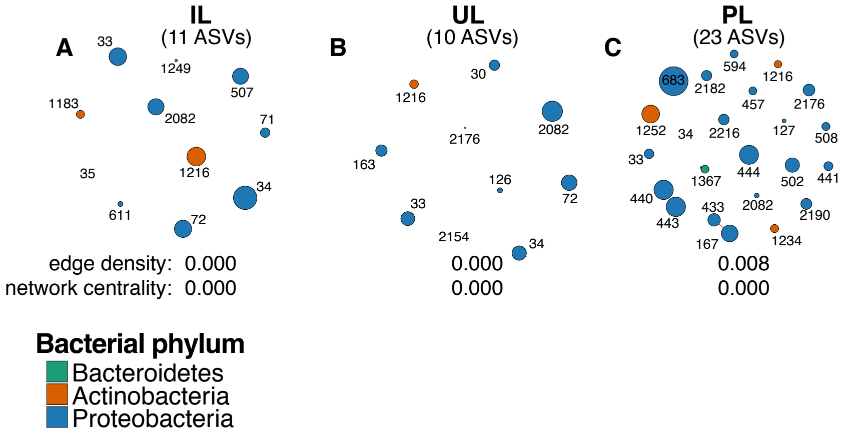

Figure S6 Leaf bacterial networks of *Orobanche* and *Hedera* inferred using SPIEC-EASI (qualitatively similar results obtained using SparCC for network inference). We inferred the bacterial network of (A) leaves of infected *Hedera,* (B) leaves of uninfected *Hedera,* and (C) leaves of *Orobanche.* Node colour and size represent bacterial phylum classification and abundance (centered log-ratio transformed), respectively. Nodes are labelled with an arbitrary number assigned to individual ASVs. Edge colour and width represent sign (dark shade = positive association, light shade = negative association), and strength of co-association, respectively.

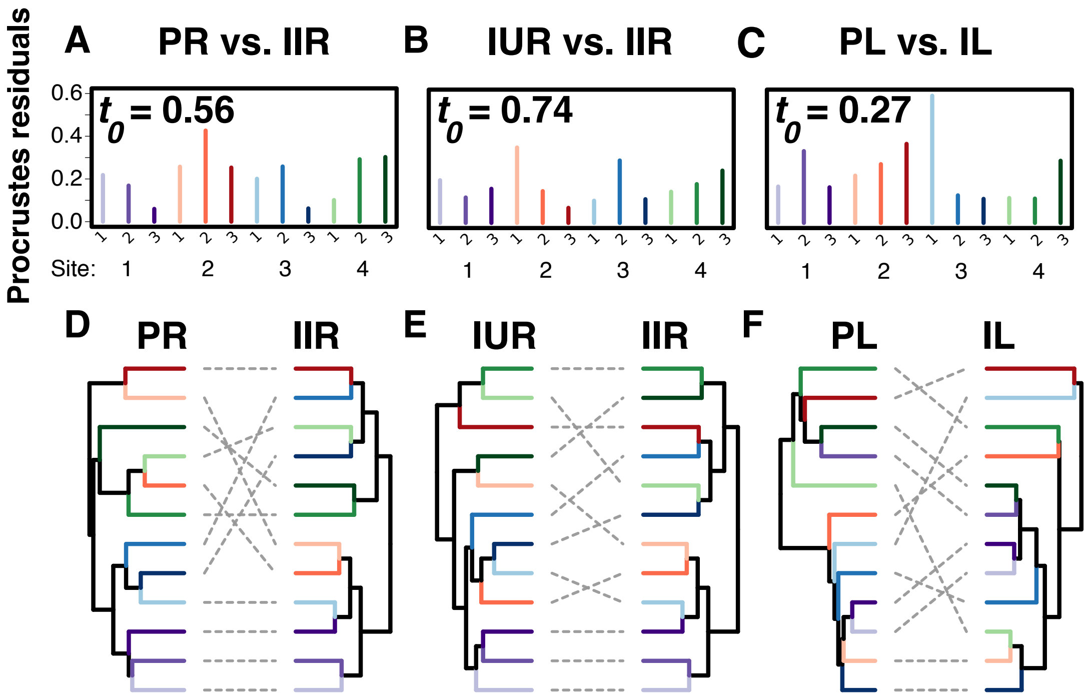

Figure S7 Procrustes residuals (A-C) and dendrograms (D-F) of select bacterial community comparisons for two pairs of communities that exhibit significant congruence (PR vs. IIR [A, D]; IUR vs. IIR [B, E]), and one pair that exhibits non-significant congruence (PL vs. IL [C, F]). Colours represent the infected host/parasite sample (three per site) from a given site (site 1 – purple; site 2 – red; site 3 – blue; site 4 – green). The Procrustes residuals represent the level of discordance between corresponding observations occurring within two bacterial communities. For example, the discordance between the bacterial community occurring within (A) *Orobanche* roots versus infected *Hedera* roots was low in infected host/parasite pair 3 from site 1 but comparatively high in infected host/parasite pair 2 from site 2. Lower residual values between corresponding bacterial communities lead to higher values of the overall Procrustes correlation-like statistic (t_0). Hierarchical clustering of individual samples within community types reflects the results obtained from the Procrustes analysis. Community types that exhibit higher Procrustes congruence (A, B) also display greater dendrogram concordance (D, E). Hierarchical clustering was performed using the weighted UniFrac distance between all samples within a community type followed by clustering by the unweighted pair group method with arithmetic mean (UPGMA). Dendrograms were created using the 'dendextend' R package version 1.9.0 (Galili, 2015).

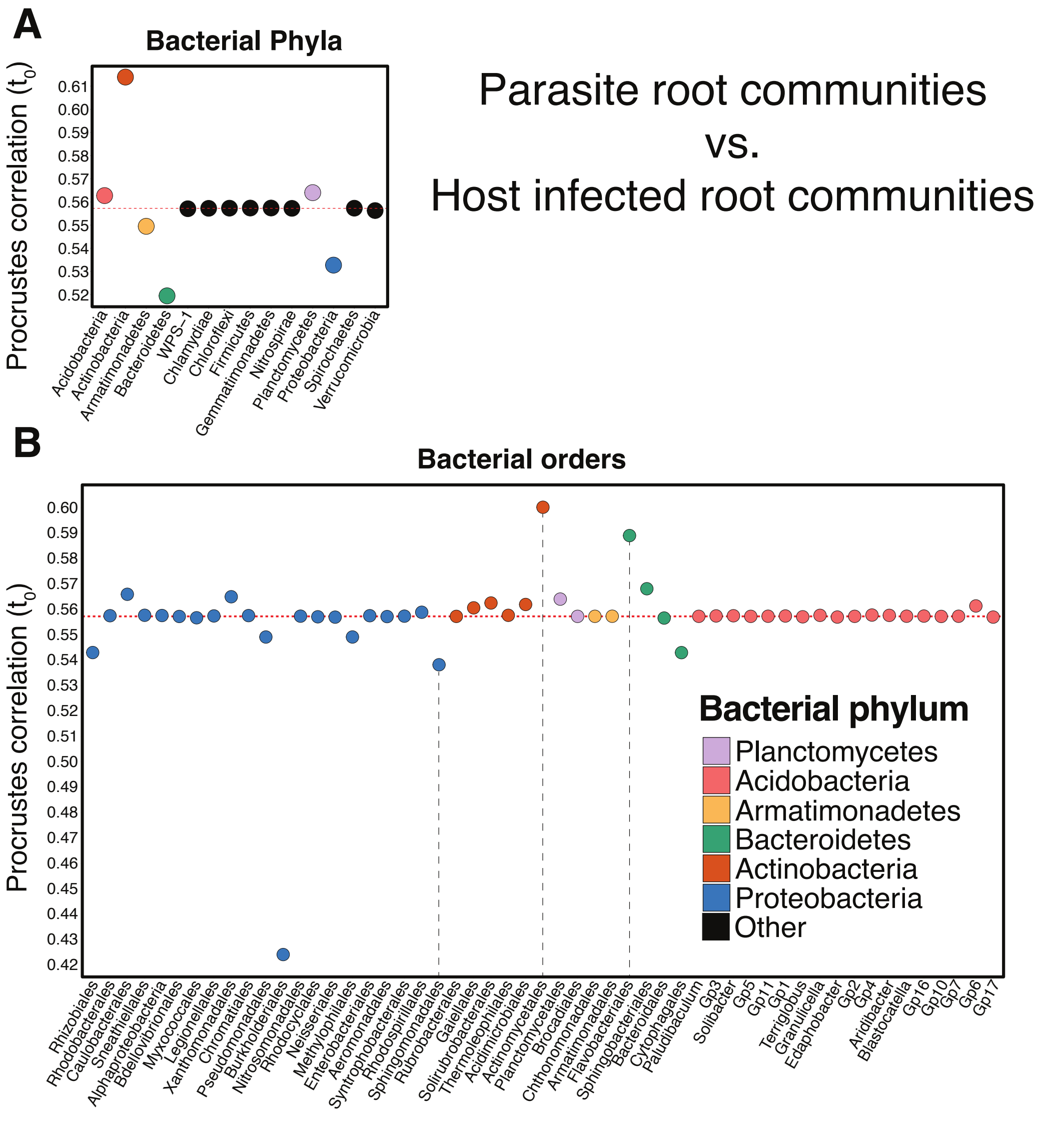

Figure S8 Our leave-one-out approach (see Materials and Methods: Procrustes tests), revealed that a subset of bacterial taxa at the level of (A) phylum or (B) order contributed strongly to the congruence or discordance between *Orobanche* and *Hedera* root communities.

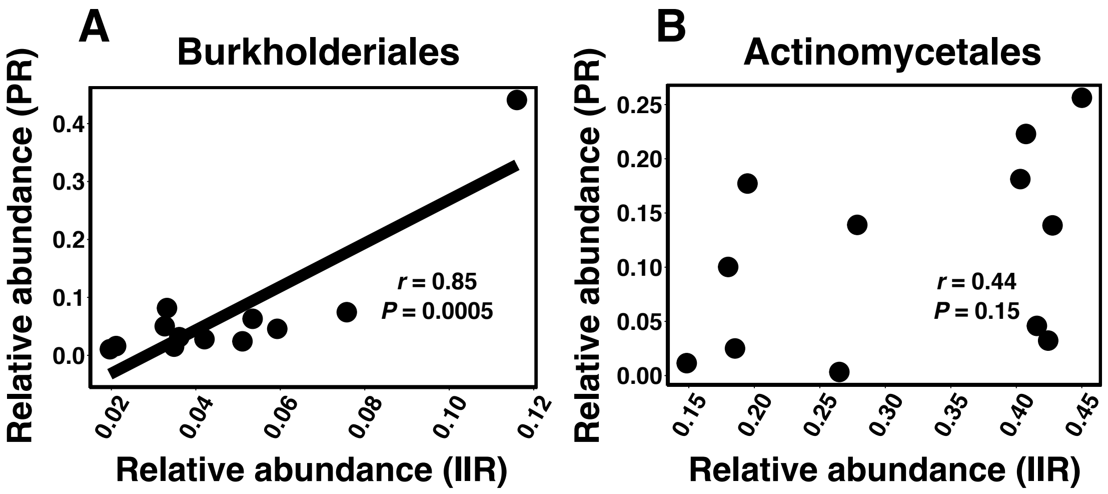

Figure S9 The relative abundance of the (A) Burkholderiales, a bacterial order that contributes to the congruence between *Orobanche* and *Hedera* root communities (Fig. S8), is highly correlated between host and parasite roots. Note this relationship is still significant even after removal of the point in the top right corner. Conversely, the relative abundance of the (B) Actinomycetales, a bacterial order that contributes to the discordance between *Orobanche* and *Hedera* root communities, is uncorrelated between host and parasite roots.

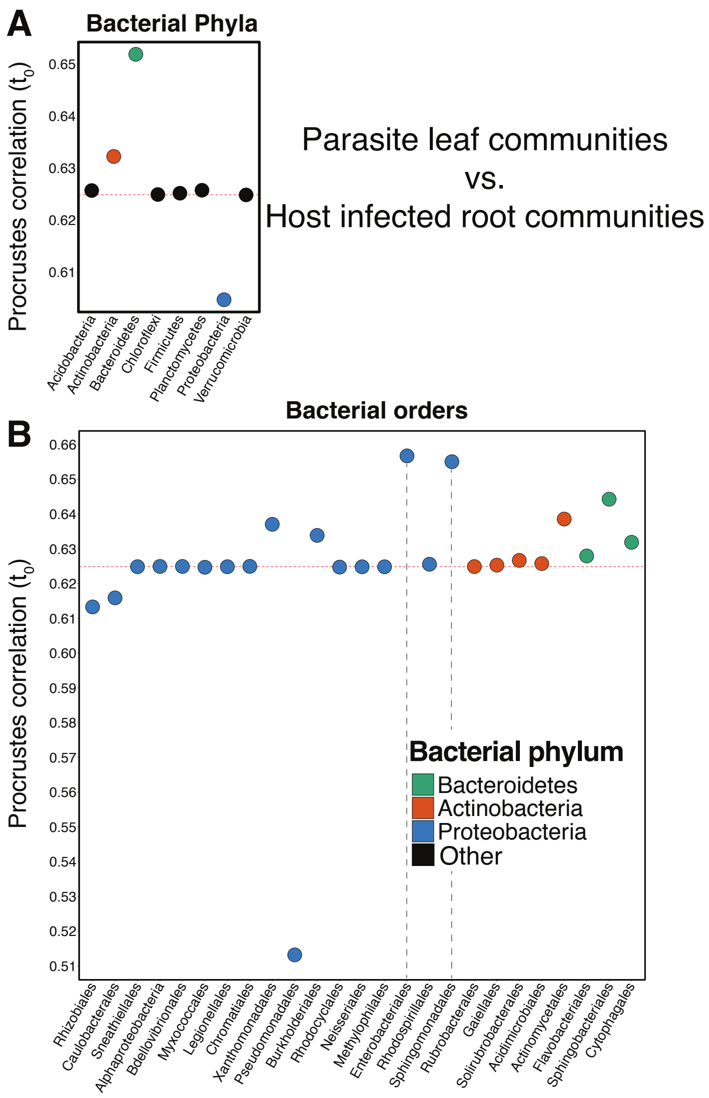

Figure S10 Our leave-one-out approach (see Materials and Methods: Procrustes tests), revealed that a subset of bacterial taxa at the level of (A) phylum or (B) order contributed strongly to the congruence or discordance between *Orobanche* leaf and *Hedera* root communities.

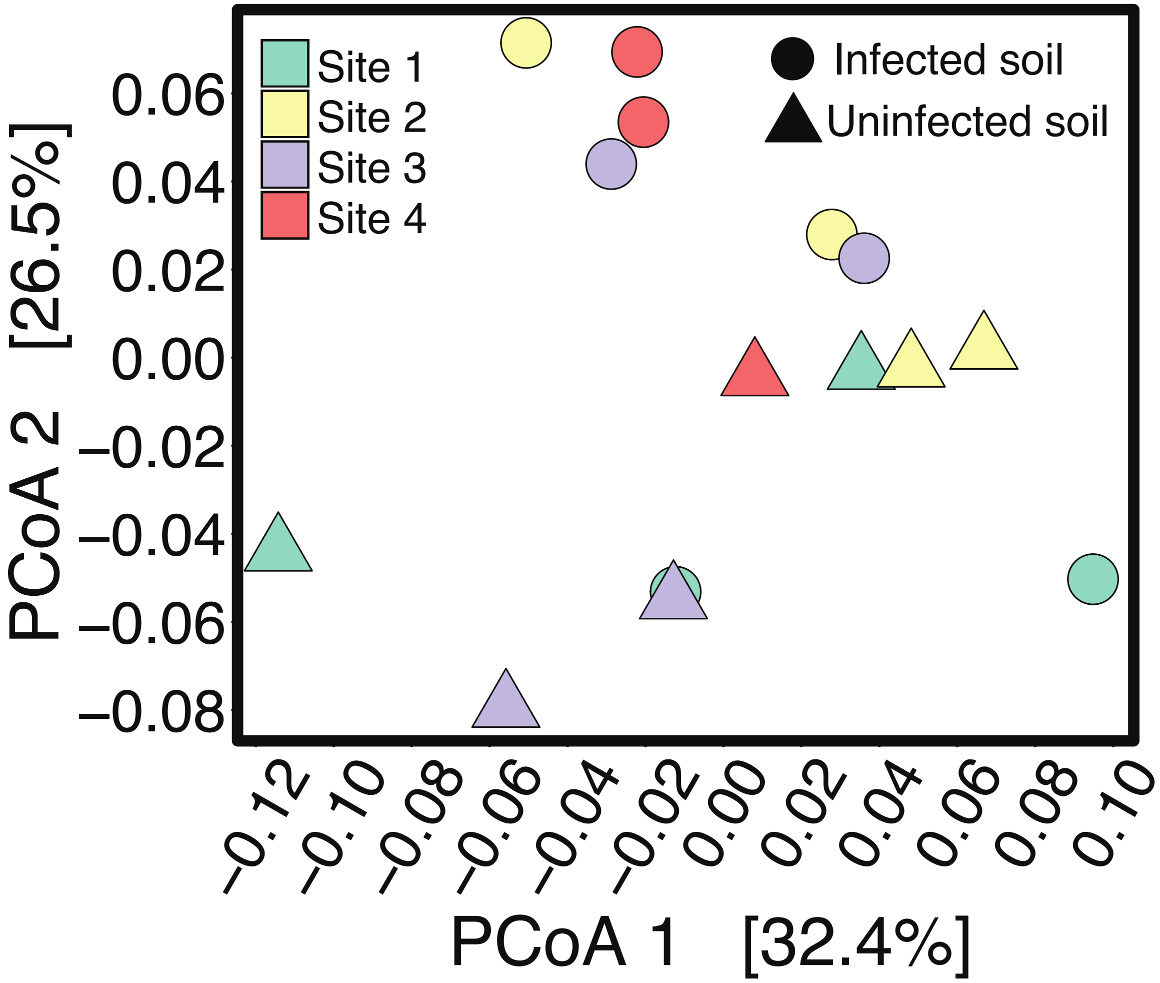

Figure S11 Principle coordinate analysis of weighted UniFrac dissimilarity among soil bacterial communities. We found no consistent effects of either infection (P=0.18) or site (P= 0.32; results from PERMANOVA).

Table S1 Genome sizes and sampling locations for each *Hedera* individual collected in the experiment. Genome size (measured using flow cytometry) and species identifications were determined by Alan Whittemore (U.S. National Arboretum). Vouchers deposited at or distributed by the United States National Arboretum Herbarium (NA). Vouchers are labeled "Schneider & Fitzpatrick" followed by the collection number indicated.

Individual	Genome size (pg)	Inferred ploidy	Species	Latitude	Longitude	Voucher collection number [b]
1.I.1	5.95	4x	*H. algeriensis* [a]	37.871500	-122.265189	--
1.I.2	5.89	4x	*H. algeriensis* [a]	37.871500	-122.265189	1046
1.I.3	5.84	4x	*H. algeriensis* [a]	37.871500	-122.265189	1047
1.U.1	6.00	4x	*H. algeriensis*	37.871572	-122.264768	--
1.U.2	5.88	4x	*H. algeriensis*	37.871572	-122.264768	1055
1.U.3	5.86	4x	*H. algeriensis* [a]	37.871572	-122.264768	--
2.I.1	3.01	2x	*H. helix*	37.873610	-122.264592	--
2.I.2	5.91	4x	*H. algeriensis*	37.873679	-122.264634	--
2.I.3	3.08	2x	*H. helix*	37.873748	-122.264630	1048
2.U.1	5.86	4x	*H. algeriensis*	37.873713	-122.264289	--
2.U.2	5.82	4x	*H. algeriensis*	37.873713	-122.264289	1056
2.U.3	6.12	4x	*H. algeriensis*	37.873713	-122.264289	1057
3.I.1	5.77	4x	*H. algeriensis*	37.872382	-122.265023	1049
3.I.2	5.95	4x	*H. algeriensis*	37.872382	-122.265023	1050
3.I.3	5.96	4x	*H. algeriensis*	37.872382	-122.265023	1051
3.U.1	5.65	4x	*H. algeriensis*	37.87239	-122.26480	1058
3.U.2	5.96	4x	*H. algeriensis*	37.87239	-122.26480	1059
3.U.3	6.07	4x	*H. algeriensis*	37.87239	-122.26480	1060
4.I.1	5.89	4x	*H. algeriensis*	37.869848	-122.261662	1052
4.I.2	5.86	4x	*H. algeriensis*	37.869848	-122.261662	1053
4.I.3	5.65	4x	*H. algeriensis*	37.869848	-122.261662	1054
4.U.1	5.96	4x	*H. algeriensis*	37.869973	-122.261492	--
4.U.2	6.03	4x	*H. algeriensis*	37.869973	-122.261492	1061
4.U.3	6.07	4x	*H. algeriensis*	37.869973	-122.261492	--

[a] Possibly introgressed with *H. hibernica*

[b] Some specimens had too little material after DNA extraction to voucher

Table S2 Linear mixed effect model results for the analysis of α-diversity. Evenness was calculated as inverse Simpson's diversity/ASV richness and phylogenetic diversity was calculated as the sum of the total phylogenetic branch length for a given sample. The top table presents the effects of plant species, organ type, and site on α-diversity measures across all communities. The bottom tables present the effects of *Hedera* infection status and site on leaf and root communities separately.

Hedera* spp. & *Orobanche hederae

	log(Observed ASV richness)		log(Simpson's diversity $^{-1}$)		Evenness		Phylogenetic diversity	
	F/X²	*P*	*F/X²*	*P*	*F/X²*	*P*	*F/X²*	*P*
Species (S)	0.01	0.91	1.15	0.31	1.92	0.19	1.62	0.23
Organ (O)	92.20	**<0.001**	17.16	**<0.001**	0.46	0.51	153.5	**<0.001**
S × O	13.40	**0.01**	5.46	0.06	0.67	0.45	18.07	**0.007**
log(UsableReads)	243.69	**<0.001**	11.15	**0.001**	35.42	**<0.001**	128.36	**<0.001**
Site (Si)	0.00	1.00	0.00	1.000	0.00	1.00	0.00	1.00
S × Si	0.00	1.00	0.65	0.42	0.22	0.64	0.00	1.00
O × Si	0.00	1.00	0.00	1.00	0.00	1.00	0.00	1.00
S × O × Si	0.00	1.00	2.56	0.11	0.01	0.92	0.00	1.00

***Hedera* spp. leaf microbiome**

	log(Observed ASV richness)		log(Simpson's diversity $^{-1}$)		Evenness		Phylogenetic diversity	
	F/X²	*P*	*F/X²*	*P*	*F/X²*	*P*	*F/X²*	*P*
Infection	0.14	0.73	0.62	0.49	0.82	0.43	0.81	0.43
log(UsableReads)	488.18	**<0.001**	66.17	**<0.001**	25.32	**<0.001**	218.56	**<0.001**
Site	0.00	1.00	0.00	1.00	0.01	0.91	0.00	1.00
Infection × site	0.00	1.00	2.56	0.11	0.99	0.32	0.00	1.00

***Hedera* spp. root microbiome**

	log(Observed ASV richness)		log(Simpson's diversity $^{-1}$)		Evenness		Phylogenetic diversity	
	F/X²	*P*	*F/X²*	*P*	*F/X²*	*P*	*F/X²*	*P*
Infection	0.62	0.48	1.00	0.36	0.71	0.44	0.04	0.85
log(UsableReads)	35.80	**<0.001**	0.26	**0.61**	3.81	0.07	23.58	**<0.001**
Site	0.00	1.00	0.00	1.00	0.00	1.00	0.02	0.88
Infection × site	2.48	0.12	0.00	1.00	0.00	1.00	4.66	**0.03**

Table S3 Linear mixed effect model results for the analysis of β-diversity. UniFrac PCoA refers to sample scores along PCoA axes using weighted UniFrac dissmilarity among samples. Bray-Curtis PCoA refers to sample scores along PCoA axes using Bray-Curtis dissmilarity among samples. The top table presents the effects of plant species, organ type, and site on β-diversity measures across all communities. The bottom tables present the effects of *Hedera* infection status and site on leaf and root communities separately.

Hedera* spp. & *Orobanche hederae

	UniFrac PCoA 1		UniFrac PCoA 2		UniFrac PCoA 3		Bray-Curtis PCoA 1		Bray-Curtis PCoA 2		Bray-Curtis PCoA 3	
	F/X^2	P	F/X^2	P	F/X^2	P	F/X^2	P	F/X^2	P	F/X^2	P
Species (S)	51.32	**<0.001**	14.85	**0.002**	7.82	**0.02**	49.39	**<0.001**	61.58	**<0.001**	6.28	**0.03**
Organ (O)	64.04	**<0.001**	3.07	0.10	4.21	**0.05**	308.75	**<0.001**	0.02	0.89	0.46	0.52
S × O	65.50	**<0.001**	0.09	0.77	11.54	**0.02**	70.96	**<0.001**	12.75	**0.01**	42.43	**0.003**
log(UsableReads)	4.93	**0.03**	0.40	0.53	2.78	0.10	9.22	**0.003**	0.23	0.63	0.03	0.86
Site (Si)	0.00	1.00	0.00	1.00	0.59	0.81	0.00	1.00	0.00	1.00	0.00	0.96
S × Si	0.00	1.00	0.00	1.00	0.00	1.00	0.00	1.00	0.31	0.58	0.00	1.00
O × Si	0.00	1.00	2.89	0.09	0.00	1.00	0.00	1.00	0.00	1.00	2.30	0.13
S × O × Si	0.00	1.00	0.00	1.00	0.00	1.00	0.12	0.73	0.00	1.00	0.07	0.80

***Hedera* spp. leaf microbiome**

	UniFrac PCoA 1		UniFrac PCoA 2		UniFrac PCoA 3		Bray-Curtis PCoA 1		Bray-Curtis PCoA 2		Bray-Curtis PCoA 3	
	F/X^2	P	F/X^2	P	F/X^2	P	F/X^2	P	F/X^2	P	F/X^2	P
Infection	0.89	0.41	0.09	0.79	1.59	0.29	0.75	0.45	0.79	0.44	0.58	0.50
log(UsableReads)	1.07	0.31	0.06	0.82	3.97	0.06	11.63	**0.003**	1.20	0.29	0.07	0.80
Site	0.00	1.00	0.00	1.00	0.00	1.00	0.00	1.00	0.00	1.00	0.04	0.83
Infection × site	0.40	0.53	0.00	1.00	0.00	1.00	0.01	0.93	0.00	1.00	0.00	1.00

***Hedera* spp. root microbiome**

	UniFrac PCoA 1		UniFrac PCoA 2		UniFrac PCoA 3		Bray-Curtis PCoA 1		Bray-Curtis PCoA 2		Bray-Curtis PCoA 3	
	F/X^2	P	F/X^2	P	F/X^2	P	F/X^2	P	F/X^2	P	F/X^2	P
Infection	0.52	0.51	3.43	0.15	0.16	0.71	0.96	0.39	1.05	0.37	0.74	0.45
log(UsableReads)	1.13	0.30	3.59	0.07	1.98	0.17	0.30	0.58	0.01	0.93	2.07	0.16
Site	0.39	0.53	0.00	1.00	0.06	0.81	0.00	1.00	0.00	1.00	0.05	0.83
Infection × site	2.03	0.15	9.12	**0.003**	3.07	0.08	6.75	**0.009**	13.24	**<0.001**	14.59	**<0.001**

Table S4 Bacterial ASVs unique to host tissues. We list the full ASV sequence as well as the prevalence in the given tissue type (see supplemental excel sheet).

Table S5 Full differential abundance. For each taxonomic rank (different coloured rows), we report the differential abundance results for each taxon across each of the six contrasts. Positive values of differential abundance indicate that a bacterial taxon was found at higher abundance in samples corresponding to the first term in the listed contrast. Differential abundance is estimated as the $\log_2$-fold change in read count (DESeq2: LFC) or as the relative difference in read count between experimental factors versus within experimental factors (ALDEx2: Effect). Both DESeq2 and ALDEx2 yielded qualitatively similar results. All *P* values were corrected using the False Discovery Rate. We also list the average relative abundance of each taxon in leaf and root tissues across both host plant species (see supplemental excel sheet).

Table S6 Network attributes of bacterial communities in *Orobanche* and *Hedera* leaves and roots using two methods, SPIEC-EASI or SparCC. Edge density is the observed proportion of the total possible associations within a network of a given size. Network centrality is the network-level measure of betweenness centrality normalized to the theoretical maximum based on network size (see Materials and Methods).

	SPIEC-EASI		SparCC	
Community	Edge density	Network centrality	Edge density	Network centrality
IIR	0.020	0.035	0.016	0.093
IUR	0.022	0.025	0.014	0.050
UR	0.021	0.019	0.017	0.058
PR	0.009	0.004	0.006	0.004
IL	0.000	0.000	0.073	0.067
UL	0.000	0.000	0.111	0.198
PL	0.008	0.000	0.004	0.000

Table S7 Results from a series of Procrustes analyses, in which individual sample scores along the first two principle coordinate axes of separate ordinations were matched between different bacterial communities. For example, in the first row of the following table the sample scores from a PCoA of IIR and a PCoA of IUR were matched with a Procrustes analysis. The fit between two sets of scores is given by the Procrustes correlation-like statistic (t_0), which ranges from 0 (no fit) to 1 (perfect fit). Numerous permutations of PCoA scores among individual samples followed by recalculation of t_0 provides a test of the statistical significance of the observed fit between two sets of PCoA scores. We also include three tests to serve as a 'sanity check', which we expect to exhibit non-significant congruence (e.g. *Orobanche* root microbiota should not exhibit any congruence with those of uninfected *Hedera*).

**Procrustes tests**			
**Ordination 1**	**Ordination 2**	**Procrustes correlation (t_0)**	***P***
IIR	IUR	0.74	**0.002**
IIR	PL	0.62	**0.02**
IUR	PL	0.61	**0.02**
IIR	PR	0.56	**0.03**
IUR	PR	0.56	**0.03**
IIR	IS	0.91	**0.04**
IUR	IS	0.91	**0.04**
PR	IS	0.95	0.08
PL	PR	0.50	0.11
IL	IUR	0.46	0.14
IL	PR	0.48	0.15
UR	UL	0.37	0.32
UR	US	0.83	0.33
PL	IS	0.66	0.38
IIR	IL	0.33	0.53
IL	IS	0.66	0.58
UL	US	0.72	0.58
IL	PL	0.27	0.66
**Sanity check**			
IIR	UR	0.31	0.61
PR	UR	0.26	0.80
PL	UR	0.27	0.68

Table S8 Results from our leave-one-out approach at the level of bacterial phylum for both parasite root and leaf microbial communities. For parasite root and leaf communities separately, we removed all bacterial ASVs from the parasite dataset classified to a given bacterial phyla, re-calculated weighted UniFrac distances among all samples, obtained sample scores from a new PCoA, and re-calculated t_0 with the original *Hedera* infected root PCoA. The effect of excluding a particular bacterial phylum on the fit between host and parasite microbiota is given by $\Delta t = (t_{excluded} - t_0)$.

Goodness of fit (t_0)	*P*	Number of ASVs excluded	Phylum	Δ t_0	Organ
0.519	0.07	129	Bacteroidetes	-0.038	root
0.533	0.06	498	Proteobacteria	-0.025	root
0.549	0.05	6	Armatimonadetes	-0.008	root
0.556	0.05	42	Verrucomicrobia	-0.001	root
0.557	0.05	5	Can. Div. WPS-1	0.000	root
0.557	0.05	1	Nitrospirae	0.000	root
0.557	0.06	1	Chlamydiae	0.000	root
0.557	0.04	1	Spirochaetes	0.000	root
0.557	0.05	10	Chloroflexi	0.000	root
0.557	0.05	3	Firmicutes	0.000	root
0.557	0.04	3	Gemmatimonadetes	0.000	root
0.563	0.05	98	Acidobacteria	0.005	root
0.564	0.04	67	Planctomycetes	0.007	root
0.614	0.01	284	Actinobacteria	0.057	root
0.605	0.02	166	Proteobacteria	-0.020	leaf
0.625	0.02	4	Verrucomicrobia	0.000	leaf
0.625	0.02	1	Chloroflexi	0.000	leaf
0.625	0.02	2	Firmicutes	0.000	leaf
0.626	0.02	12	Acidobacteria	0.001	leaf
0.626	0.01	6	Planctomycetes	0.001	leaf
0.632	0.02	62	Actinobacteria	0.007	leaf
0.652	0.01	31	Bacteroidetes	0.027	leaf

Table S9 Results from our leave-one-out approach at the level of bacterial order for both parasite root and leaf microbial communities. For parasite root and leaf communities separately, we removed all bacterial ASVs from the parasite dataset classified to a given bacterial order, re-calculated weighted UniFrac distances among all samples, obtained sample scores from a new PCoA, and re-calculated t_0 with the original *Hedera* infected root PCoA. The effect of excluding a particular bacterial phylum on the fit between host and parasite microbiota is given by $\Delta t = (t_{excluded} - t_0)$.

Goodness of fit (t_0)	*P*	Number of ASVs excluded	Order	Phylum	Δ t_0	Organ
0.424	0.22	55	Burkholderiales	Proteobacteria	-0.133	root
0.538	0.06	34	Sphingomonadales	Proteobacteria	-0.019	root
0.543	0.06	46	Cytophagales	Bacteroidetes	-0.014	root
0.543	0.06	140	Rhizobiales	Proteobacteria	-0.014	root
0.549	0.06	12	Pseudomonadales	Proteobacteria	-0.008	root
0.549	0.05	4	Methylophilales	Proteobacteria	-0.008	root
0.557	0.05	1	Bacteroidales	Bacteroidetes	-0.001	root
0.557	0.04	41	Myxococcales	Proteobacteria	-0.001	root
0.557	0.04	1	Neisseriales	Proteobacteria	0.000	root
0.557	0.05	5	Gp17	Acidobacteria	0.000	root
0.557	0.06	3	Edaphobacter	Acidobacteria	0.000	root
0.557	0.05	2	Terriglobus	Acidobacteria	0.000	root
0.557	0.05	5	Rhodocyclales	Proteobacteria	0.000	root
0.557	0.05	1	Aeromonadales	Proteobacteria	0.000	root
0.557	0.05	3	Bdellovibrionales	Proteobacteria	0.000	root
0.557	0.06	2	Gp10	Acidobacteria	0.000	root
0.557	0.05	1	Rubrobacterales	Actinobacteria	0.000	root
0.557	0.05	1	Candidatus_Brocadiales	Plancotmycetes	0.000	root
0.557	0.04	1	Gp5	Acidobacteria	0.000	root
0.557	0.05	2	Gp2	Acidobacteria	0.000	root
0.557	0.04	1	Chthonomonadales	Armatimonadetes	0.000	root
0.557	0.05	1	Paludibaculum	Acidobacteria	0.000	root
0.557	0.05	3	Gp7	Acidobacteria	0.000	root
0.557	0.04	1	Armatimonadales	Armatimonadetes	0.000	root
0.557	0.05	1	Gp11	Acidobacteria	0.000	root
0.557	0.04	1	Blastocatella	Acidobacteria	0.000	root
0.557	0.06	1	Nitrosomonadales	Proteobacteria	0.000	root
0.557	0.05	2	Syntrophobacterales	Proteobacteria	0.000	root
0.557	0.03	14	Gp16	Acidobacteria	0.000	root
0.557	0.04	2	Gp1	Acidobacteria	0.000	root
0.557	0.04	2	Legionellales	Proteobacteria	0.000	root
0.557	0.05	2	Gp3	Acidobacteria	0.000	root
0.557	0.05	5	Candidatus_Solibacter	Acidobacteria	0.000	root
0.558	0.05	5	Rhodobacterales	Proteobacteria	0.000	root
0.558	0.05	3	Enterobacteriales	Proteobacteria	0.000	root
0.558	0.05	3	Chromatiales	Proteobacteria	0.000	root
0.558	0.06	9	Alphaproteobacteria	Proteobacteria	0.000	root
0.558	0.05	5	Aridibacter	Acidobacteria	0.000	root
0.558	0.06	2	Granulicella	Acidobacteria	0.000	root

Table S9 continued

Goodness of fit (t_0)	P	Number of ASVs excluded	Order	Phylum	Δ t_0	Organ
0.558	0.04	3	Thermoleophilales	Actinobacteria	0.000	root
0.558	0.06	3	Sneathiellales	Proteobacteria	0.000	root
0.558	0.05	4	Gp4	Acidobacteria	0.001	root
0.559	0.05	51	Rhodospirillales	Proteobacteria	0.002	root
0.561	0.04	19	Gaiellales	Actinobacteria	0.003	root
0.561	0.04	40	Gp6	Acidobacteria	0.004	root
0.562	0.04	40	Acidimicrobiales	Actinobacteria	0.005	root
0.563	0.04	37	Solirubrobacterales	Actinobacteria	0.005	root
0.564	0.04	65	Planctomycetales	Plancotmycetes	0.007	root
0.565	0.05	53	Xanthomonadales	Proteobacteria	0.008	root
0.566	0.04	14	Caulobacterales	Proteobacteria	0.009	root
0.568	0.04	58	Sphingobacteriales	Bacteroidetes	0.011	root
0.589	0.03	21	Flavobacteriales	Bacteroidetes	0.032	root
0.600	0.03	161	Actinomycetales	Actinobacteria	0.043	root
0.513	0.09	12	Pseudomonadales	Proteobacteria	-0.112	leaf
0.613	0.02	51	Rhizobiales	Proteobacteria	-0.012	leaf
0.616	0.03	6	Caulobacterales	Proteobacteria	-0.009	leaf
0.625	0.02	11	Myxococcales	Proteobacteria	0.000	leaf
0.625	0.03	1	Rhodocyclales	Proteobacteria	0.000	leaf
0.625	0.02	1	Neisseriales	Proteobacteria	0.000	leaf
0.625	0.02	1	Methylophilales	Proteobacteria	0.000	leaf
0.625	0.02	1	Legionellales	Proteobacteria	0.000	leaf
0.625	0.02	1	Sneathiellales	Proteobacteria	0.000	leaf
0.625	0.01	1	Rubrobacterales	Actinobacteria	0.000	leaf
0.625	0.02	2	Alphaproteobacteria	Proteobacteria	0.000	leaf
0.625	0.02	1	Bdellovibrionales	Proteobacteria	0.000	leaf
0.625	0.02	1	Chromatiales	Proteobacteria	0.000	leaf
0.625	0.01	3	Gaiellales	Actinobacteria	0.000	leaf
0.626	0.01	10	Rhodospirillales	Proteobacteria	0.001	leaf
0.626	0.01	4	Acidimicrobiales	Actinobacteria	0.001	leaf
0.627	0.02	6	Solirubrobacterales	Actinobacteria	0.002	leaf
0.628	0.02	6	Flavobacteriales	Bacteroidetes	0.003	leaf
0.632	0.02	12	Cytophagales	Bacteroidetes	0.007	leaf
0.634	0.02	18	Burkholderiales	Proteobacteria	0.009	leaf
0.637	0.01	14	Xanthomonadales	Proteobacteria	0.012	leaf
0.639	0.01	46	Actinomycetales	Actinobacteria	0.014	leaf
0.644	0.02	13	Sphingobacteriales	Bacteroidetes	0.019	leaf
0.655	0.01	23	Sphingomonadales	Proteobacteria	0.030	leaf
0.657	0.01	5	Enterobacteriales	Proteobacteria	0.032	leaf
